# Optimized culture methods for isolating small extracellular vesicles derived from human induced pluripotent stem cells

**DOI:** 10.1002/jev2.12065

**Published:** 2021-04-10

**Authors:** Ying Luo, Dunqin Gao, Peng Wang, Cheng Lou, Tong Li, Wenhui Niu, Yingtang Gao

**Affiliations:** ^1^ Tianjin Key Laboratory of Artificial Cell Tianjin Institute of Hepatobiliary Disease Nankai University Affiliated Third Center Hospital Tianjin China; ^2^ Tianjin Medical University Third Center Clinical College Tianjin China; ^3^ Department of Hepatobiliary Surgery Nankai University Affiliated Third Center Hospital Tianjin China; ^4^ Department of Cardiology Tianjin Key Laboratory of Artificial Cell Nankai University Affiliated Third Center Hospital Tianjin China

**Keywords:** extracellular vesicles‐production medium, human induced pluripotent stem cells (hiPSCs), pluripotency, small extracellular vesicles

## Abstract

Extracellular vesicles that are derived from stem cells play an important role in the treatment of disease. To obtain high‐quality small extracellular vesicles (sEVs), we optimized the culture conditions of human induced pluripotent stem cells (hiPSCs), the supernatant collection time, and sEVs extraction methods. Firstly, hiPSCs were cultured in extracellular vesicles‐production medium (EVs‐PM) containing different concentrations (0%, 0.25%, 0.5%, 2%, 5%, and 20%) of extracellular vesicle‐depleted knockout serum replacement (ED‐KSR), and the culture supernatants were collected continuously for 5 days. Then, the sEVs were isolated, followed by an evaluation of their characteristics. The survival rates of the hiPSCs lines that were cultured in EVs‐PM containing 0.5% to 20% ED‐KSR were not significantly different (*P *> 0.05). The survival rates of the hiPSCs in 0.5% ED‐KSR after the culture supernatants were continuously collected for day 1, day 3, and day 5 were not statistically significant (*P *> 0.05). After 5 days of continuous collection of the supernatant, the hiPSCs expressed some pluripotent markers, while SSEA4 and TRA‐1‐60 expression changed gradually. The sEVs that were extracted by the two methods were all 50–200 nm, double‐layered and oval or round cellular vesicles and expressed the marker proteins CD63, TSG101, and HSP70. The characteristics of sEVs extracted on day 1, day 3, and day 5 were almost identical on morphology, size and the relative quantity of annexin V‐positive subpopulations. The PKH67 staining showed that the sEVs could be endocytosed by HepG2 cells and aggregated in the cytoplasm. The proliferation experiments showed that the sEVs can promote cell proliferation. In Conclusion, the 0.5% ED‐KSR is the optimal concentration, and that the hiPSCs culture supernatant can be continuously collected for 5 days while maintaining high cell viability and some pluripotent characteristics. Both of the methods extraction can be used to obtain biologically active sEVs.

## 1 BACKGROUND

The reprogramming of somatic cells, which eventually leads to the formation of human induced pluripotent stem cells (hiPSCs), was demonstrated in 2007 (Takahashi et al., 2007). The hiPSCs are not only similar to embryonic stem cells (ESCs) in their cell morphology and surface antigens, and they also have a multi‐directional differentiation function and infinite proliferative ability (Yu et al., 2007). Moreover, hiPSCs have been induced to differentiate into a variety of mature somatic cells in vitro (Chin et al., 2009); their application in the clinical setting (Amabile & Meissner, 2009) will relieve the moral and ethical problems of the use of embryonic stem cells (Volarevic et al., 2018), and hiPSCs are becoming increasingly popular in the field of regenerative medicine. However, to achieve clinical applicability, the problem of tumour formation in hiPSCs needs to be overcome (Ratajczak et al., 2014).

Extracellular vehicles (EVs) are released by most viable cells through paracrine effects and play a pivotal role in inter‐cellular communication due to their ability to actively transport various functional proteins, mRNAs, microRNAs and lipids (Colombo et al., 2014, Yeung et al., 2018). In recent years, some researchers have found that EVs are linked to disease progression and cancer metastasis (Hendrix & Hume, 2011, Alderton, 2012). EVs that are derived from hiPSCs have also been identified to have prominent therapeutic potential in diverse types of disease (Taheri et al., 2019). Therefore, several studies have focused on the biological characteristics and extraction methods of EVs derived from hiPSCs. According to Minimal information for studies of extracellular vesicles 2018 (MISEV2018), small extracellular vehicles (sEVs) are an appropriate term that can be used to describe these vesicles, especially when a method is used to select for vesicles smaller than 200 nm without determining their intracellular origin (Théry et al., 2018). In this study, we use sEVs instead of exosomes, because the isolated EVs are vesicles with a diameter smaller than 200 nm and are extracted by the method of differential ultracentrifugation with 220 nm filtration.

To extract the hiPSCs‐derived sEVs, a suitably extraction medium must be prepared that will ensuring the pluripotency of hiPSCs and enable the collection of cellular supernatants and the extraction of biologically active sEVs (Zhu et al., 2017). Previous literature has described the conditioned media required for the collection of sEVs from various cellular sources, such as conditioned media with low serum adaptation or serum‐free media (Gao et al., 2016, Ye et al., 2014). However, very little research has been devoted to the specific discussion of the extraction medium required to collect sEVs from iPSCs. When collecting sEVs from hiPSCs, it is important to extract the biologically active sEVs and optimize the culture medium to minimize the effects of sEVs while maintaining the pluripotency of the iPSCs. Therefore, we chose knockout serum replacement (KSR) (Thermo Fisher Scientific, Carlsbad, CA, USA), which is a serum replacement with a defined nutrient composition (Wagner & Welch, 2010). In addition, during the sEVs extraction, we optimized and used two methods: the traditional ultracentrifugation method and the ExoQuick‐TC kit (EQ) (System Bioscience, Palo Alto, CA, USA) to extract and purify sEVs, and to determine whether the culture supernatant collected from the hiPSCs under the above conditions can extract biologically active sEVs.

In this study, we used two lines of hiPSCs as sEVs donor cells to identify the best culture conditions for collecting the hiPSCs culture supernatants. Additionally, using modified ultracentrifugation (M‐UC) and the EQ to extract sEVs, we verified whether the extracted sEVs had biological activity, thus laying the foundation for follow‐up studies of sEVs.

## 2 METHODS

### 2.1 Preparation of EV‐depleted knockout serum replacement (ED‐KSR) and extracellular vesicles‐production medium (EVs‐PM)

The EVs‐PM should first be prepared to reduce the impact of other proteins on sEVs. We chose a fetal bovine serum replacement known as KnockOut™ Serum Replacement (KSR, Cat. No. 10828028, Thermo Fisher Scientific, Carlsbad, CA, USA), which is a defined, FBS‐free medium supplement that supports the growth of hiPSCs cultured on feeder culture dishes. To extract sEVs, the EVs or EVs‐like particles that may exist in the KSR need to be removed; therefore, EVs‐depleted knockout serum replacement (ED‐KSR) was obtained by ultracentrifugation of the KSR at 120,000 × *g* for 18 h at 4°C using a P40ST rotor (CP80MX, Hitachi, Japan) (Baietti et al., 2012). Thus, the ED‐KSR provided less interference to the sEVs so that a certain concentration of ED‐KSR could then be added to D/F12 medium (Cat. No. 01‐172‐1ACS, Biological Industries, Beit Haemek, Israel) to prepare the EVs‐PM for the culture of hiPSCs for collection of the cellular supernatants.

### 2.2 hiPSCs culture and collection of EVs‐PM supernatant

Two hiPSCs lines were used for extraction of sEVs (hiPSCs‐EC1 and hiPSCs‐HF1); hiPSCs‐EC1 were purchased from Beijing Cellapy Biotechnology Co., Ltd. (Cat. CA4024106, Beijing, China), and were induced from the urine of renal epithelial cells using *Oct4, Sox2, Klf4*, and *c‐myc* (Suppl. File 1, and http://c1629707695.bj.wezhan.cn/a5) (Luo et al., 2018). hiPSCs‐HF1 were kindly provided by Dr. Sui Zhang (Department of Cardiology, The University of Texas MD Anderson Cancer Center, Houston, Texas, USA) and were induced from IMR‐90 human fibroblasts using *Oct4, Sox2, Klf4*, and *c‐myc* (Figure S1). Under normal conditions, hiPSCs‐EC1 were cultured in E8 complete medium (STEMCELL Technologies, Vancouver, B.C., Canada) and the hiPSCs‐HF1 were cultured in mTeSR™1 complete medium (STEMCELL Technologies, Vancouver, B.C., Canada) at 37℃ in humidified air with 5% CO_2_. It is worth noting that culture dishes or plates should be coated with Matrigel Matrix (BD Biosciences, New Jersey, USA) for 24 h before use. The mycoplasma (Biotool, USA) and endotoxin detection (Beijing Jinshanchuan, China) were negative in the two hiPSCs lines (Figure S2).

When 1×10^6^ hiPSCs‐EC1 or hiPSCs‐HF1 reached a density of 70%–80% in a 6‐well culture plate, the mTeSR™1 medium was removed and replaced with 2 ml of EVs‐PM per well. First, to identify the optimal ED‐KSR concentration, hiPSCs‐EC1 and hiPSCs‐HF1 were cultured in EVs‐PM containing 20%, 5%, 2%, 0.5%, 0.25%, and 0% ED‐KSR, and the culture supernatants were collected after 24 h. The control group consisted of hiPSCs that were cultured under normal conditions. Cell morphology was monitored by phase contrast microscopy (DMIL LED, Leica, Germany). The FITC Annexin V Apoptosis Detection Kit (BD Biosciences, New Jersey, USA) and flow cytometry were used to determine the viability of each group of cells. SDS‐PAGE was used to observe the protein expression in the sEVs as well as the effects of protein impurities, which were assessed to determine the optimal concentration of ED‐KSR to use for the study.

Second, we determined the optimum collection time for the culture supernatants. At the best ED‐KSR concentration, the culture supernatant was collected every day and replaced with freshly EVs‐PM. The pluripotency and survival rate of the hiPSCs at the times of collection (day 1, day 3, and day 5) were detected, the viability of each group of cells was determined by flow cytometry, and Alkaline phosphatase, immunofluorescence, flow cytometry, and qRT‐PCR were used to verify the pluripotency of each group of cells.

### 2.3 Extraction of sEVs

**FIGURE 1:**
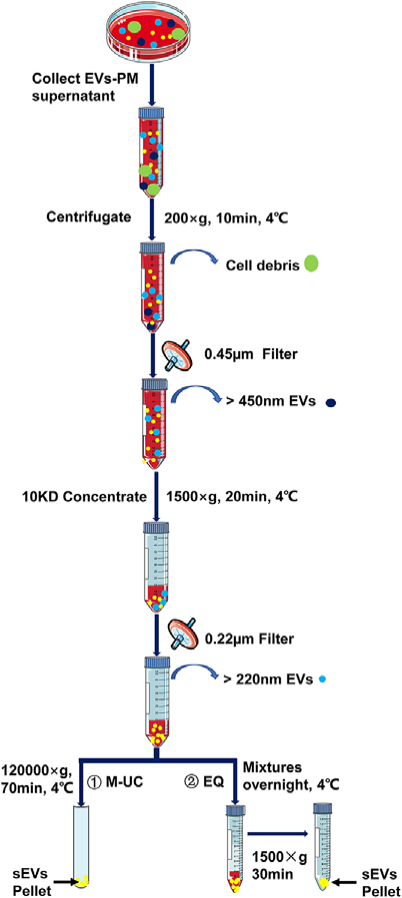
Schematic of exosome purification using the two methods. ① modified ultracentrifugation; ② the ExoQuick kit from the SBI company

First, the collected 16 ml of EVs‐PM was centrifuged at 200 × *g* for 10 min, the supernatant was filtered through a 0.45 μm filter, and the filtrate was concentrated using a 10‐KD molecular weight cut‐off (MWCO) hollow fiber membrane (Merck Millipore, Darmstadt, Germany) at 1500 × *g* for 30 min. On average, 16 ml of medium was concentrated to 200–400 μl and then filtered through a 0.22 μm filter, to exclude all particles larger than 200 nm. The filtrates were then treated with two different methods to extract sEVs (Figure 1).

For the modified ultracentrifugation (M‐UC) method, the 200 μl of the filtrates were diluted to 10 ml in PBS, transferred to a 13 ml centrifuge tube (13PA Tube, Hitachi, Japan) and ultracentrifuged at 120,000 × *g* for 70 min at 4°C using a P40ST rotor (CP80MX, Hitachi, Japan). Then the pellet was resuspended in 20 μl of sterile PBS and stored at −80℃ for future use.

For the ExoQuick‐TC kit (EQ) (System Bioscience, Palo Alto, CA, USA) method, the filtrates and ExoQuick‐TC solution were mixed by inversion or flicking in a sterile tube at a 5:1 ratio. The mixtures were refrigerated overnight for at least 12 h at 4℃, and care was given to ensure that the tubes remained upright and did not rotate during the incubation period. On the second day, the mixtures were centrifuged at 1500 × *g* for 30 min, followed by aspiration of the supernatant. The centrifugation and aspiration steps were repeated (the former for 5 min), and the pellet was then resuspended in PBS and stored at −80℃ for future use.

### 2.4 Flow cytometry detection

Cell apoptosis was evaluated using the FITC Annexin V Apoptosis Detection Kit I according to the manufacturer's instructions (BD Biosciences, New Jersey, USA). First, 10 × Binding Buffer was diluted to a 1 × concentration with deionized water; then, the adherent cells were digested with EDTA‐free trypsin at room temperature. The digestates were centrifuged at 400 × *g* for 5 min, and 1 × 10^6^ cells were then collected, resuspended and washed in 1 × PBS by centrifugation at 400 × *g* for 5 min at 4°C. Next, 300 μl of 1 × Binding Buffer was added to resuspend the cells, and 3.5 μl of Annexin V‐FITC and 3.5 μl of Propidium Iodide (PI) were added to label cells, which were then incubated in the dark for 15 min at room temperature. Next, 200 μl of 1 × Binding Buffer was added before running the sample on the BD‐FACS Canto II Flow Cytometer (BD Biosciences, New Jersey, USA). Acquired data was analysed with FlowJo 7.6 software.

#### 2.4.1 Detection of pluripotent markers

The hiPSCs‐EC1 after supernatant collection for 1, 3, and 5 days were dissociated in 0.05% trypsin‐ethylenediaminetetraacetic acid and washed twice in PBS. 1 × 10^6^ cells were incubated with 20 μl of the solution mouse anti‐human SSEA4 antibody, mouse anti‐human TRA‐1‐60 antibody (Abcam, Cambridge, UK. Table S1) or the isotype controls for 30 min. Cells were washed twice in 1 ml PBS and resuspended in 500 μl PBS. Flow cytometric analysis was performed with BD FACSCanto II Flow Cytometer (BD Biosciences, New Jersey, USA). Acquired data was analysed with FlowJo 7.6 software.

Relative quantity analysis of sEVs subpopulations based on Annexin V: 1 μl of hiPSCs‐EC1‐sEVs resuspension was diluted to 100 μl in PBS, stained with FITC‐labelled annexin V (BD Biosciences, New Jersey, USA) for 30 min at room temperature. Then the 100 μl of resuspension was diluted to 1 ml in PBS. Flow cytometry was performed using a CytoFLEX flow cytometer (Beckman Coulter, USA). The EVs events were gated as 50–200 nm.

### 2.5 Alkaline phosphatase detection

Alkaline phosphatase was detected using the BCIP/NBT alkaline phosphate esterase colour kit (Beyotime, Shanghai, China), which includes an alkaline phosphatase colour developing buffer, a BCIP reagent and an NBT reagent. First, the hiPSCs were fixed in 4% paraformaldehyde for 1.5 min. The BCIP/NBT working solution was prepared according to the manufacturer's instructions and then mixed with the fixed hiPSCs to fully cover the cell sample. The mixtures were protected from light and incubated for 5–30 min. Then, the BCIP/NBT working solution was removed, and cells were washed three times with PBS, after which they were observed under an Olympus IX71 fluorescence microscope (Olympus, Tokyo, Japan).

### 2.6 Immunofluorescence detection

hiPSCs were fixed in 4% paraformaldehyde for 30 min, permeabilized with 0.5% Triton X‐100, and incubated overnight at 4℃ with primary antibodies against Oct‐4, SSEA4, and SOX2 purchased from Abcam (Cambridge, UK. Table S1). Following day, an Alexa Fluor 488‐conjugated anti‐mouse IgG secondary antibody (Thermo Fisher Scientific, Carlsbad, CA, USA) was used for the detection of SSEA4, and an Alexa Fluor 546‐conjugated anti‐rabbit IgG secondary antibody (Thermo Fisher Scientific, Carlsbad, CA, USA) was used for the detection of Oct‐4 and SOX2. Finally, the nuclei were stained with DAPI. After they were mounted, the samples were observed under an Olympus IX71 fluorescence microscope and images were taken by DP70 (Olympus Corporation, Japan). The images were analysed with Image‐Pro Plus 5.1 (Media Cybernetics, Singapore).

### 2.7 Transmission electron microscopy (TEM)

10 μl of the purified sEVs were adsorbed to a copper mesh, left at room temperature for 1 min, and then stained with phosphotungstic acid (pH 6.8) for 5 min at room temperature; afterwards, the sample was dried under a lamp. The samples were then imaged using a Hitachi HT7700 transmission electron microscope (Hitachi, Tokyo, Japan). The test was completed by the public service platform for large instruments of Tianjin Medical University.

### 2.8 Nanoparticle tracking analysis (NTA)

2 μl of the sEVs sample was diluted with 500 μl PBS to the required concentration and injected onto the Malvern Panalytical Nanosight LM10 instrument (Malvern Panalytical, Malvern, UK) with a 1 ml syringe, and the analysed data were then saved. The test was conducted by Malvern Panalytical (Shanghai, China).

### 2.9 Dynamic Light Scattering (DLS)

The average particle dimensions and concentration of the sEVs were characterized by the DLS technique (ZetaView, Particle Metrix). 2 μl of the sEVs sample was diluted with 500 μl PBS to the required concentration. The test was conducted by OBiO Technology (Shanghai) Corp., Ltd.

### 2.10 SDS‐PAGE analysis of the total sEVs proteins contents

The sEVs (20 μl) were used for SDS‐PAGE analysis, taking D/F12 (20 μl), ED‐KSR (1 μl ED‐KSR+19 μl H_2_0), and 0.5% EVs‐PM (20 μl) as control. All samples were mixed with 5X loading buffer (Beyotime, Shanghai, China) and then loaded in equal volume, followed by heating to 100°C for 5 min for denaturation, and the samples were then loaded onto a 10% SDS‐polyacrylamide gel (Beyotime, Shanghai, China) in equal volumes, and run at 80 V for 30 min, and the gel was separated at 120 V for 1 h. Then SDS‐PAGE gels were stained using Coomassie Blue. The gels were imaged using a FluorChem™ M system (ProteinSimple, San Jose, California).

### 2.11 Western blotting analysis of the iPSCs and sEVs

The total proteins content in the iPSCs was extracted by a RIPA kit (Beyotime, Shanghai, China), and the protein samples of the sEVs were detected directly without extracted by a RIPA kit. The total amount of protein was detected by bicinchoninic acid method (BCA). All the samples were loaded in equal amount (15 μg), and then separated by a 10% SDS‐polyacrylamide gel, transferred to a polyvinylidene difluoride membrane (Beyotime, Shanghai, China). The membrane was blocked with 5% bovine serum albumin in TBST (Beyotime, Shanghai, China) and incubated overnight with primary antibodies. The blots were then washed with TBST, incubated with anti‐rabbit or anti‐mouse secondary antibodies (Cat. No. S0001 and S0002. Affinity Bioscience, OH, USA) and detected with the ECL‐2 reagent (Beyotime, Shanghai, China). The primary antibodies that were used in this study were anti‐HSP70, anti‐CD63, anti‐TSG101, anti‐calreticulin, and anti‐β‐actin which were all purchased from System Biosciences (SBI, Palo Alto, CA, USA) or Abcam (Cambridge, UK). Detailed information of antibodies was shown in the Table S1.

### 2.12 PKH67‐labeled sEVs and coculture with HepG2 cells

The membrane dye PKH67 is commonly used to label sEVs. According to the manufacturer's instructions, 500 μl of Diluent C and 25 μl of sEVs were first mixed to prepare Liquid A; then, 500 μl of Diluent C and 3 μl of PKH67 were prepared to form Liquid B, which was then mixed with Liquid A and incubated at room temperature for 1–5 min, this mixture was then neutralized with 5 ml of PBS and filtered on a 0.22 μm filter, and PKH67‐labeled sEVs were then extracted by ultracentrifugation. Next, the PKH67‐labeled sEVs were added to HepG2 cells and cultured for 48 h at 37˚C in humidified air with 5% CO2. After incubation, the cells were fixed with 4% paraformaldehyde solution, and the staining of the nuclei was performed with DAPI. Finally, the cells were visualized under an Olympus IX71 inverted fluorescence microscope.

### 2.13 Proliferation experiment of HepG2 cells

HepG2 cells were synchronized at G1/S phase by a double thymidine block, the synchronized HepG2 cells were then digested, seeded at a density of 6 × 10^4^ cells/ml and cultured in a 12‐well plate containing 0.5% FBS in D/F12 medium. After 24 h, the cells were attached, and different concentrations (20 μg/ml, 10 μg/ml, and 6 μg/ml) of sEVs were added to the culture medium and placed in an IncuCyte Zoom Live‐Cell Analysis System (Essen Bioscience) for culture and observed for 72 h continuously in real time. HepG2 cells that were cultured normally in a culture medium containing 0.5% FBS were used as the control group. The confluence degree of the different groups of HepG2 cells was measured at 0 h, 12 h, 24 h, 48 h, and 72 h. The time point at which the sEVs were added was noted as 0 h, while all of the other points were normalized to 0 h.

The sEVs (20 μg/ml) extracted on day 1, day 3, and day 5 were also performed the proliferation experiment on HepG2 cells. The blank sEVs extract medium (Matrigel + 0.5% or 20% ED‐KSR+D/F12 cell‐free, 37℃, 1 day) was used as control.

### 2.14 RNA extraction and quantitative reverse transcription polymerase chain reaction (qRT‐PCR)

Total RNA was extracted from the iPSCs, sEVs, and HepG2 cells using the TRIzol Reagent (Thermo Fisher Scientific, Carlsbad, CA, USA), and 2 μg of RNA (0.5 μg of sEVs) was converted to cDNA using GoScript™ Reverse Transcriptase (Promega, Madison, WI, USA) according to the manufacturer's instructions. Quantitative real‐time PCR was performed in a 25 μl reaction volume containing 12.5 μl 2X SYBR Green Premix Ex Taq Master Mix (Takara, Dalian, China), 10 pmol of primer and 1 μl of cDNA on the ViiA 7 Real‐Time PCR System (Thermo Fisher Scientific, Carlsbad, CA, USA). Each qRT‐PCR cycle consisted of denaturation for 5 min at 95°C, followed by 40 cycles at 94°C for 30 s and 1 min at 60°C. Triplicate reactions were performed for each sample. A detailed list of the specific gene primers is provided in Table 1. GAPDH was used as an internal control. Relative expression levels were calculated using the 2−ΔΔCt method (Livak & Schmittgen, 2001). The amplification efficiencies of the target and reference genes were approximately equal. A fold change between 0.5 and 2.0 meant no difference between the two groups. A fold change > 2.0 corresponded to up‐regulation, and a fold change < 0.5 corresponded to down‐regulation.

**TABLE 1:** List of primers used for qRT‐PCR

Gene name	Accession no.	Exon position	Nucleotide position (nt)	Primer sequence (5′‐3′)	Size (bp)
NANOG	NM_024865	1/2^a^	344‐366	F:AGATGCCTCACACGGAGACTGTC	131
		2	474‐451	R:CTTTTTTGCGACACTCTTCTCTGC	
OCT4	NM_002701	2/3	578‐601	F:GGTTCTATTTGGGAAGGTATTCAG	162
		3/4	739‐716	R:AGGGTTTCTGCTTTGCATATCTCC	
SOX2	NM_003106	1^b^	1440‐1464	F:GGAAATGGGAGGGGTGCAAAAGAGG	149
		1	1588‐1564	R:TGCGTGAGTGTGGATGGGATTGGTG	
GAPDH	NM_002046	8	889‐909	F:GGGCATCCTGGGCTACACTGA	143
		8/9	1031‐1006	R:CAAATTCGTTGTCATACCAGGAAATG	

^a^ The primer spans the junction of exons 1 and 2.

^b^ The human SOX2 gene is a single‐exon gene.

### 2.15 Statistical analysis

The mean values within a group were compared using one‐way analysis of variance, and inter‐group differences were assessed using least significant difference (LSD) test with SPSS Statistics ver. 22. Values of *P *< 0.05 were considered significant. All experiments were analysed at least in triplicate.

## 3 RESULTS

### 3.1 An ED‐KSR concentration of 0.5% is appropriate for hiPSCs culture

The KSR with or without UC treatment was added to D/F12 medium to prepare extraction medium containing 0.5% ED‐KSR, 20% ED‐KSR, 0.5% KSR, or 20% KSR. And then these extraction media were all performed according to the extraction method of sEVs. The BCA and DLS was detect total protein amount and number of particles. After UC treatment, the number of particles of 20% ED‐KSR in D/F12 medium was reduced to about 10% before treatment, and 0.5% ED‐KSR in D/F12 medium could not even detect particles, while the total amount of protein was no significant change (Table 2). Thus, before preparation of EVs‐PM, the KSR need to UC treatment for removing EVs or EVs‐like particles.

**TABLE 2:** The BCA and DLS analysis of KSR before and after UC treatment

	0.5% ED‐KSR	20% ED‐KSR	0.5% KSR	20% KSR
Protein concentration (mg/ml)	3.28 ± 0.18	15.93 ± 1.85	3.36 ± 0.66	36.00 ± 1.50
Particles concentration (particles/ml)	Not detected	5.91×10^8^ ± 1.0×10^7^	4.93×10^8^ ± 1.15×10^7^	7.47×10^9^ ± 1.53×10^8^
Particles diameter (nm)	Not detected	150.1 ± 2.85	166.37 ± 5.08	154.33 ± 3.54

**FIGURE 2:**
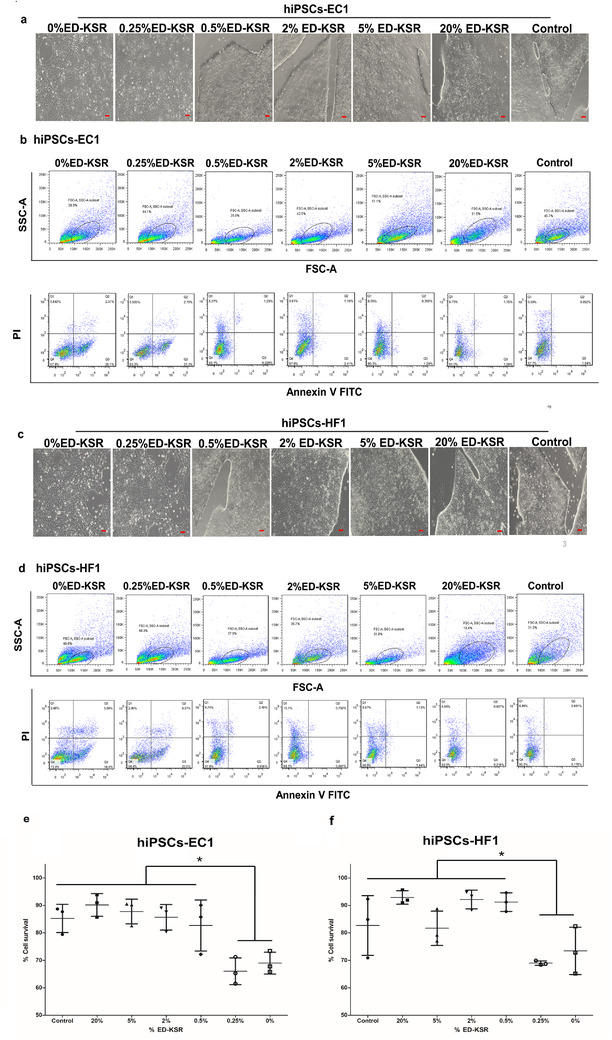
Determination of the optimal ED‐KSR concentration in extracellular vesicles‐production medium (EVs‐PM). The mTeSR™1 was replaced with fresh extraction medium containing 0%, 0.25%, 0.5%, 2%, 5%, and 20%, ED‐KSR. (a,b) The cell morphology and survival rates of hiPSCs‐EC1 after 24 h of continuous culture are shown. Bar = 50 μm. (c,d) The cell morphology and survival rates of hiPSCs‐HF1 after 24 h of continuous culture are shown. The control group consists of cells cultured in complete medium respectively. (e,f) The statistical analysis of cell survival rates of hiPSCs‐EC1 and hiPSCs‐HF1. Error bars are mean values ± SD of *n* = 3 experiments. Statistical differences were determined by one‐way analysis of variance and least significant difference (LSD) test of inter‐group differences, ^*^ *P* < 0.05

hiPSCs‐EC1 and hiPSCs‐HF1 were cultured in EVs‐PM containing 0%, 0.25%, 0.5%, 2%, 5%, and 20% ED‐KSR for 24 h, and the cell morphology and survival rate were measured. After 24 h of culture, the two hiPSCs in EVs‐PM containing 0.5%, 2%, 5%, and 20% ED‐KSR could maintain their characteristic undifferentiated morphology. Compared with the control group, the cell viabilities of the four groups (0.5%, 2%, 5%, and 20%) of two cells decreased slightly, but the difference was not significant (*P* > 0.05). However, the morphology of hiPSCs in EVs‐PM containing 0% and 0.25% ED‐KSR has changed and differentiated; the cell viabilities was also significantly decrease than the control group (Figure 2a‐f).

**FIGURE 3:**
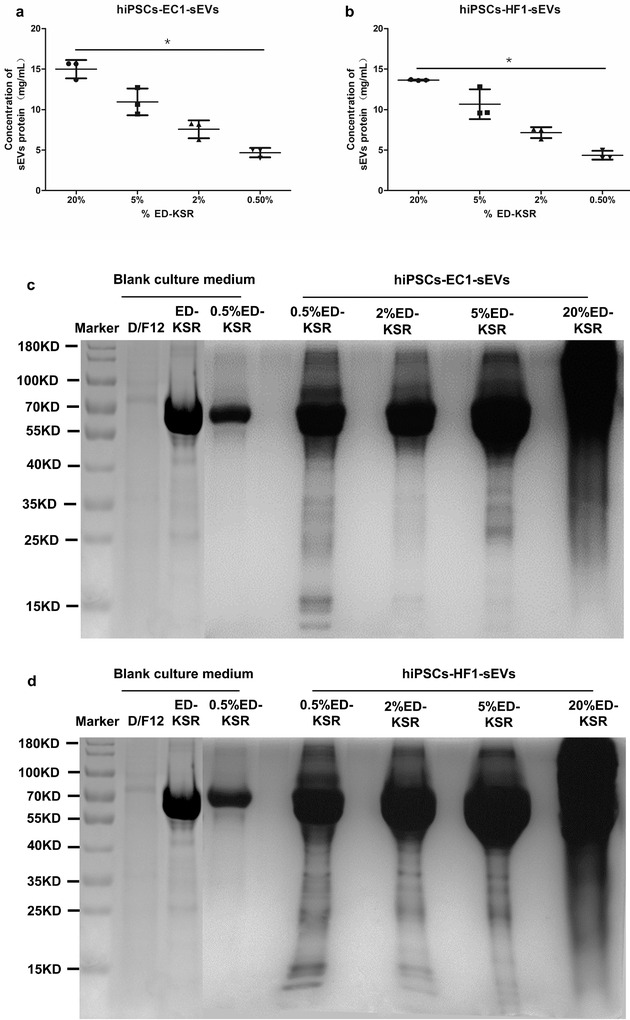
The protein concentration and distribution of sEVs samples. (a,b) The protein concentrations of the four sEVs samples derived from hiPSCs‐EC1 and hiPSCs‐HF1 (*P* < 0.05). (c,d) Comparisons of the protein content in sEVs samples and in the D/F12 and ED‐KSR media, 0.5% EVs‐PM was taken as the control. The Coomassie blue staining results are shown

The total protein concentration of the four sEVs (M‐UC) in the two cells types was determined by the BCA method and indicated that the sEVs protein concentration was significantly decreased as the ED‐KSR concentration decreased (*P* < 0.05) (Figure 3a,b). Additional SDS‐PAGE analyse showed that the most abundant protein in ED‐KSR was approximately 70 kD in size. Comparing the protein bands of the four sEVs, the 70 kD protein band became stronger with an increased ED‐KSR concentration, while the protein bands (sEVs‐specific bands) of other sizes were clearly visible in the four groups and did not change as much with the three ED‐KSR concentrations of 5%, 2%, and 0.5% (Figure 3c,d). Therefore, it was speculated that there was minimal interference between the extraction of sEVs and subsequent experiments with EVs‐PM containing 0.5% ED‐KSR.

These results indicate that 0.5% ED‐KSR does not affect the growth of hiPSCs, and that there is little protein interference from ED‐KSR on sEVs at this concentration. Thus, 0.5% ED‐KSR was selected as the best concentration to prepare EVs‐PM for collecting sEVs.

### 3.2 Cell culture supernatants can be continuously collected for 5 days

**FIGURE 4:**
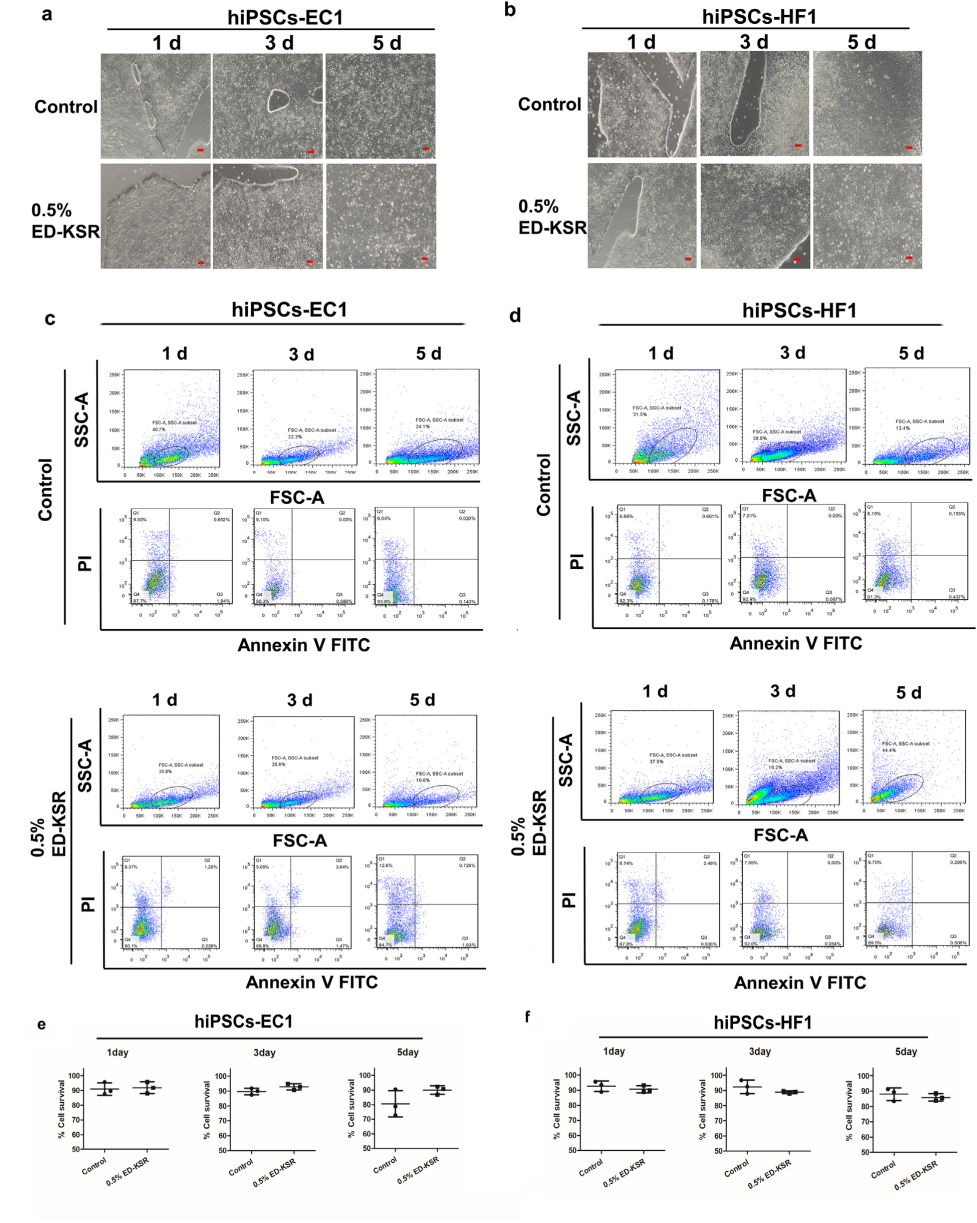
The morphology and survival rates of hiPSCs in different collect time. (a,b) The cell morphology of hiPSCs on day 1, day 3, and day 5. Bar = 50 μm. (c,d) The survival rates of two hiPSCs cell types were measured by flow cytometry. (e,f) The statistical analysis of survival rates. The control group was measured as the survival rate of hiPSCs cultured in complete medium

Fresh EVs‐PM was changed every day and the EVs‐PM supernatant was collected continuously for 5 days, then the cells after supernatant collection for 1, 3, and 5 days were selected for cell viability and pluripotency analyses. As shown in Figure 4a,b, the two lines of hiPSCs could retain their typical morphology when they were cultured in EVs‐PM containing 0.5% ED‐KSR on day 1 and day 3. However, on day 5, several cells began to appear morphological changes and became smaller, especially for hiPSCs‐HF1. Flow cytometry analysis also found that the fluorescence values of FSC and SSC generally decreased for hiPSCs‐HF1, which means that the whole cell population has shifted (Figure 4c,d). There was only a slight difference in cell viability between the three time points, and statistical analysis showed that the differences were not statistically significant (*P *> 0.05) (Figure 4e,f).

**FIGURE 5:**
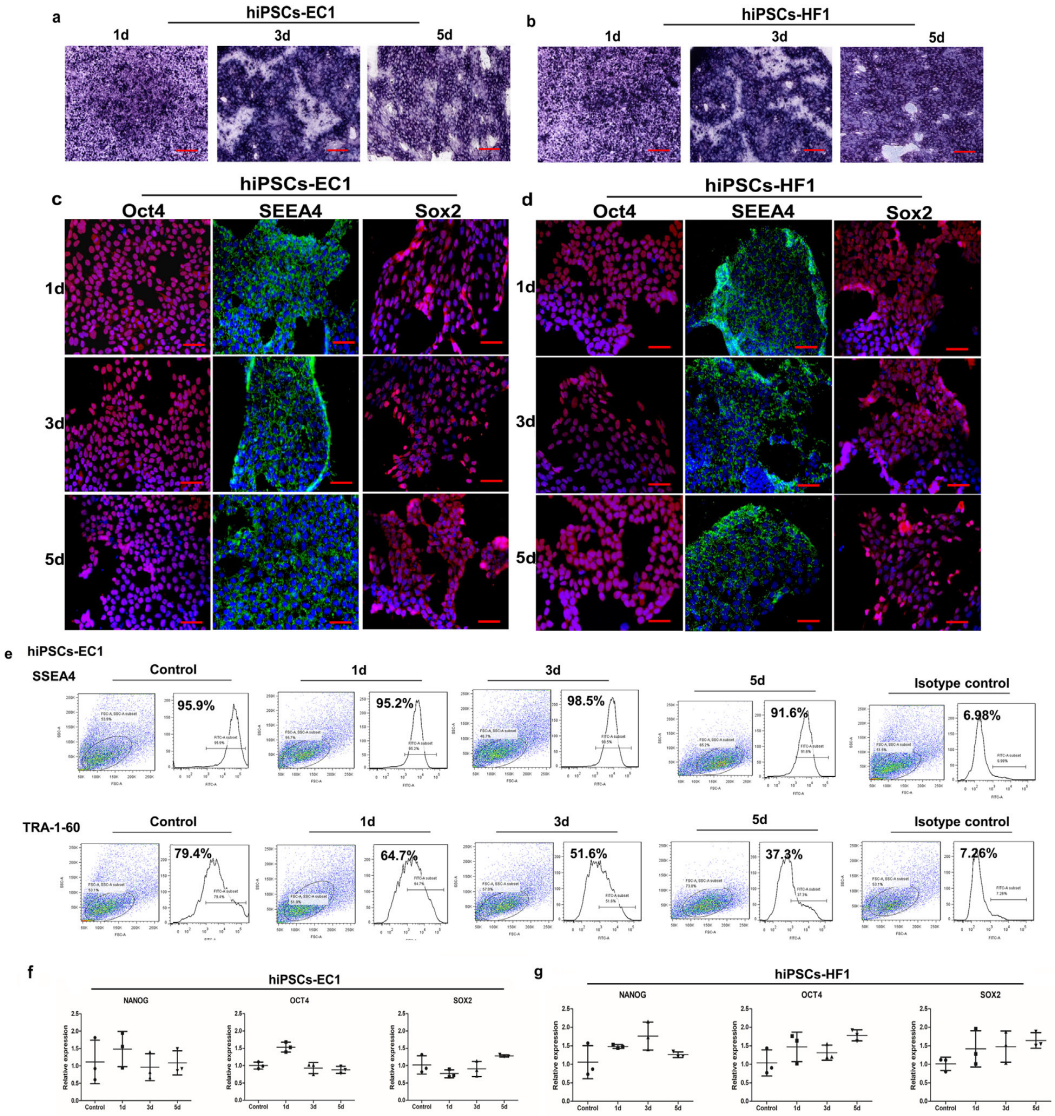
The pluripotent state of hiPSCs after continuous collection for day 1, day 3, and day 5. The culture supernatant was collected and replaced with fresh extraction medium containing 0.5% ED‐KSR. Then, the iPSCs pluripotent state after continuous collection for day 1, day 3, and day 5 were determined. (a,b) ALP staining results for hiPSCs‐EC1 and hiPSCs‐HF1. Bar = 50 μm. (c,d) Immunofluorescence results for hiPSCs‐EC1 and hiPSCs‐HF1. The figures are DAPI‐merged images. Bar = 50 μm. (e) Flow cytometry results of SSEA4 and TRA‐1‐60 for hiPSCs‐EC1. (f,g) The gene expression levels of pluripotency markers were measured by qRT‐PCR in each group of hiPSCs. The hiPSCs cultured in complete medium were used as the control

ALP activity is often used as a marker of iPSCs pluripotency; as shown in Figure 5a,b, hiPSCs expressed ALP activity after 5 days of continuous collection, and this figure also shows that hiPSCs were still in an undifferentiated state at these time points. For hiPSCs‐EC1 and hiPSCs‐HF1, the Oct4, SEEA4, and SOX2 proteins were still expressed after continuous collection of the culture supernatant for 5 days, indicating that hiPSCs still maintained pluripotency during this time (Figure 5c,d; Figure S3). Flow cytometry was used for analysing the hiPSCs‐EC1 expressed SSEA4 and TRA‐1‐60 for 5 days in EVs‐PM containing 0.5% ED‐KSR. As Figure 5e shown, the percentage of SSEA4 positive cells can be maintained more than 90% for 5 days, while the fluorescence intensity of SSEA4 declined gradually. In addition, the percentage of cells expressing TRA‐1‐60 reduced significantly for 5 days (from 79.4% in the control to 37.3% on day 5) (Figure 5e). Furthermore, qRT‐PCR analysis of the pluripotency markers showed that the two hiPSCs lines expressed Nanog, Oct4 and SOX2 mRNA for 5 days in EVs‐PM containing 0.5% ED‐KSR and that the fold change was less than 2, in contrast to the hiPSCs‐EC1 or hiPSCs‐HF1 under normal conditions (Figure 5f,g; Figure S4).

These results indicate that after continuous collection of the supernatant for 5 days, hiPSCs can still survive and retain a part of the pluripotent makers. Therefore, in EVs‐PM containing 0.5% ED‐KSR, the culture supernatant can be continuously collected for 5 days for the extraction of sEVs.

### 3.3 Both M‐UC and EQ can extract sEVs from hiPSCs

**FIGURE 6:**
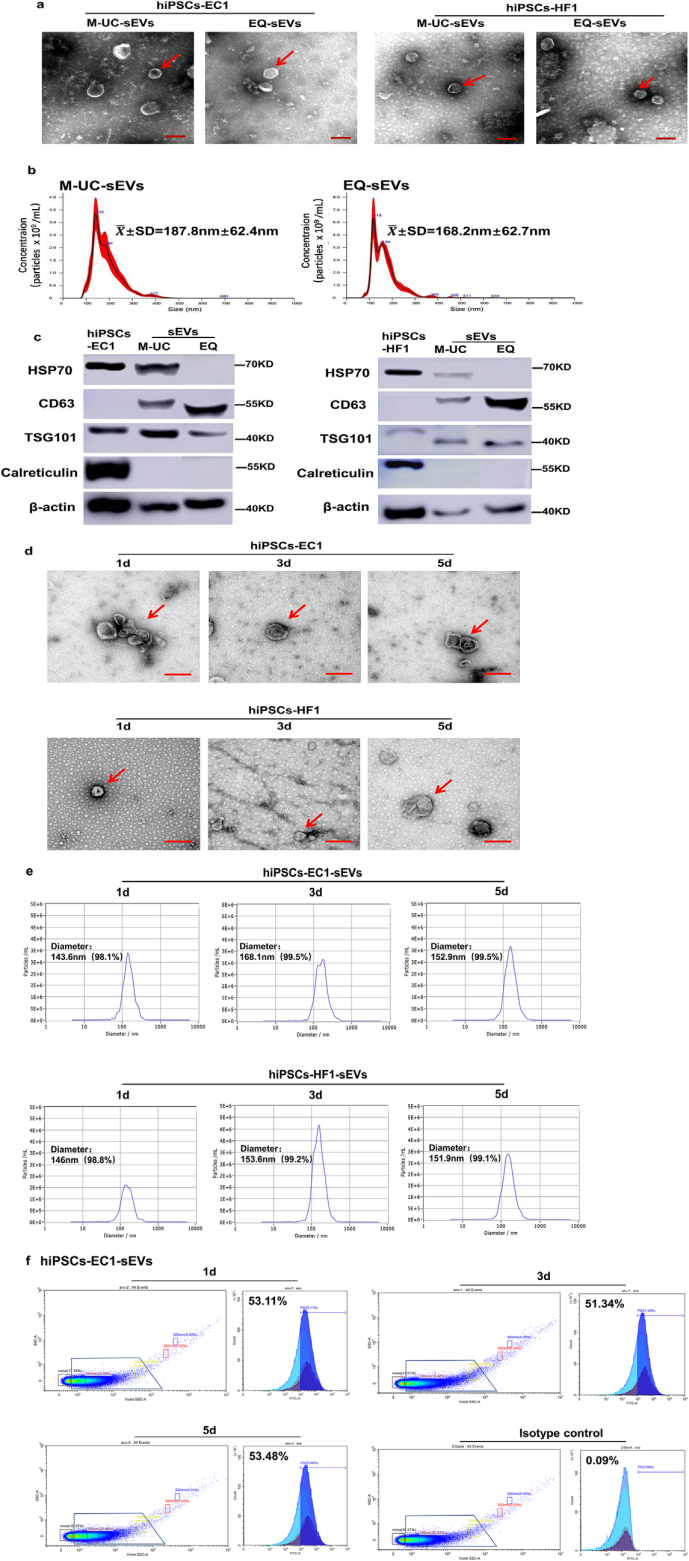
Identification of the characteristics of sEVs. (a‐c) Transmission electron microscopy (TEM), nanoparticle tracking analysis (NTA) and western blotting were used to examine the characteristics of sEVs continuously collected for 5 days. (d‐f) TEM, dynamic light scattering (DLS) and CytoFLEX flow cytometer were used to examine the characteristics of sEVs extracted on day 1, day 3, and day 5. (a) TEM results of sEVs. M‐UC‐ex represents sEVs extracted by M‐UC; EQ‐ex represents the sEVs extracted by EQ. Bar = 200 nm. (b) NTA results of sEVs derived from hiPSCs‐EC1. (c) The western blotting results. HSP70, CD63, and TSG101 are the exosome marker proteins; Calreticulin is the negative control. (d) TEM results of sEVs. Bar = 200 nm. (e) DLS results of sEVs. (f) CytoFLEX flow cytometer results of sEVs derived from hiPSCs‐EC1

To further verify the condition of the collected hiPSCs culture supernatants, we applied M‐UC and EQ to extract sEVs, and we detected the characteristics of sEVs via TEM, NTA, and WB. TEM analysis showed that sEVs that were secreted by both hiPSCs‐EC1 and hiPSCs‐HF1 had a double membrane structure with an oval or circular shape and a size of approximately 50–200 nm, as shown in Figure 6a. NTA showed that the average size of the sEVs extracted by modified ultracentrifugation was 187.8 ± 62.4 nm, and that the average size of the sEVs extracted by EQ was 168.2 ± 62.7 nm (Figure 6b; Figure S5).From the WB analysis, the sEVs marker proteins HSP70, TSG101, and CD63 were expressed in both cells and sEVs, whereas the negative control calreticulin was not expressed in the sEVs samples (Figure 6c; Figure S6). These results are as expected and show that both methods can extract sEVs. In addition, CD63 was expressed at a low level in cell lysates and was highly enriched in sEVs, which shows that sEVs may enrich proteins in cells. HSP70 was expressed only in the sEVs extracted via the modified ultracentrifugation method and not in the sEVs extracted via the EQ method.

Furthermore, the characteristics of sEVs extracted on day 1, day 3, and day 5 was detected by TEM, DLS, and flow cytometry. The sEVs extracted from the two hiPSCs lines on day 1, day 3, and day 5 were characterized by a typically heterogeneous EVs population consisting a double membrane structure and a size of approximately 50–200 nm (Figure 6d). DLS revealed the diameter was about 150 nm with more than 98% of particles extracted on day 1, day 3, and day 5 derived from two hiPSCs lines (Figure 6e). And the CytoFLEX flow cytometry analyses of annexin V was about 50% positive in the sEVs extracted on day 1, day 3, and day 5 (Figure 6f). The results showed that the relative quantity of annexin V‐positive sEVs subpopulations extracted on day 1, day 3, day 5 were almost identical .We speculated the sEVs extracted from hiPSCs cultured in sEVs‐PM have a certain consistency for 5 days.

These results indicate that both M‐UC and EQ can extract sEVs from hiPSCs‐EC1 and hiPSCs‐HF1; these results verified that the continuous 5d collection of the culture supernatant in CM containing 0.5% ED‐KSR is appropriate.

### 3.4 sEVs can enter HepG2 cells and promote their proliferation

**FIGURE 7:**
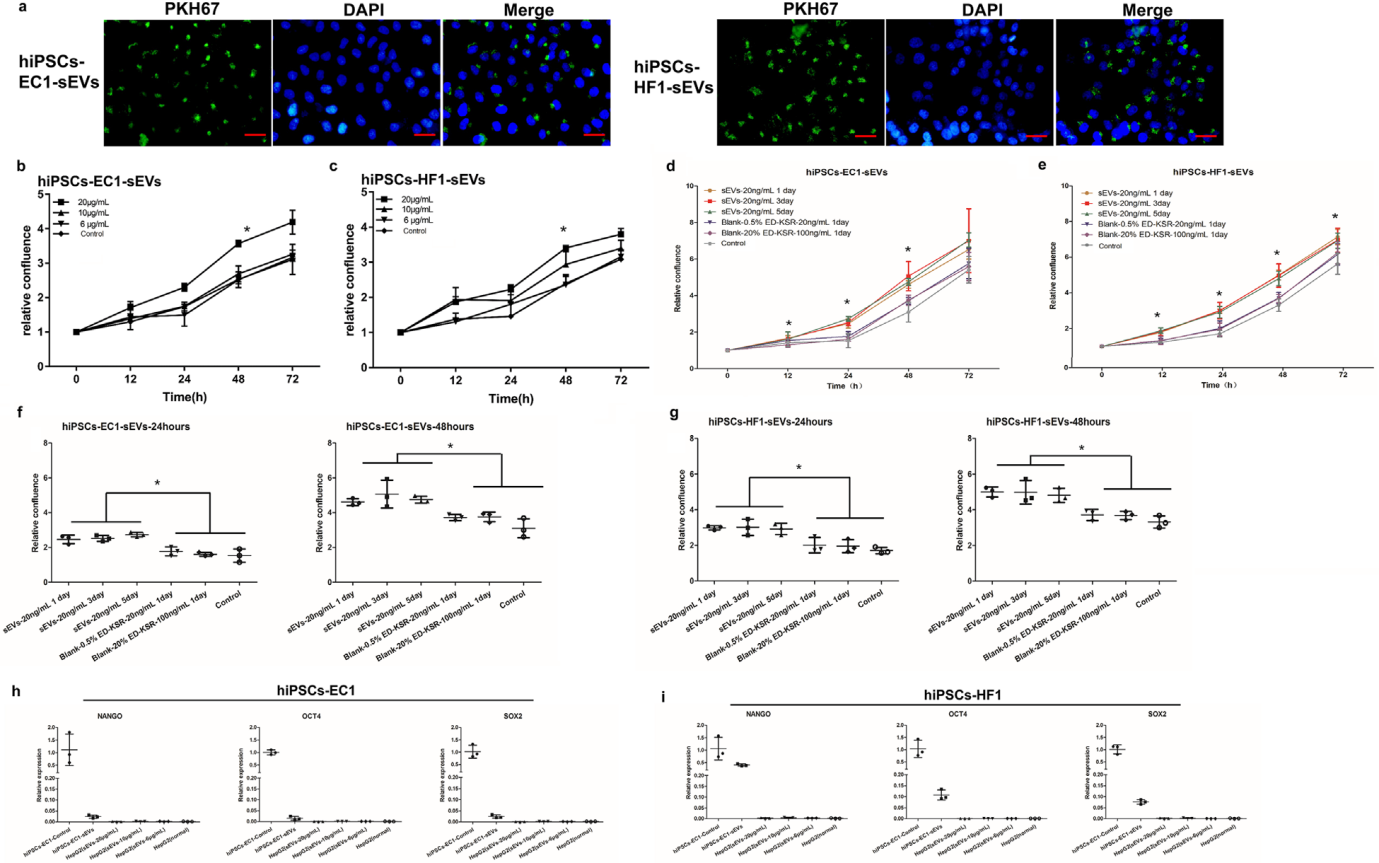
sEVs can promote the proliferation of HepG2 cells. (a) The PKH67‐sEVs were co‐cultured with HepG2 cells and HepG2 cells were found to be able to endocytose sEVs. Bar = 50 μm. (b,c) Different concentrations of sEVs continuously collected for 5 days were added to the culture medium to observe their effects on cell proliferation. Growth curves of HepG2 cells at different time points are shown. (The data were normalized by using 0 h as 1 and comparing the cell confluence degree at different time points with 0 h to calculate the relative confluence degree, and then a statistical analysis of the data was conducted.) ^∗^ *P* < 0.05. (d,e) Proliferation of HepG2 cells treated with the 20 ng/ml of sEVs extracted on day 1, day 3, and day 5. (f,g) The relative confluence degree of HepG2 cells treated with the 20 ng/ml of sEVs extracted on day 1, day 3, and day 5 at 24 h and 48 h. (h,i) The gene expression levels of pluripotency markers were measured by qRT‐PCR in sEVs and HepG2 cells that were treated with different concentrations of sEVs. The hiPSCs cells cultured in complete medium were taken as control

As shown in Figure 7a, after co‐culture of PKH67‐labeled sEVs and HepG2 cells, green fluorescence appears in the cytoplasmic and accumulates in the form of particles around the nucleus. By observing the uptake of sEVs by HepG2 cells at different time points, the sEVs were shown to enter HepG2 cells at 24 h, and the accumulation of sEVs in the HepG2 cells reached saturation at 48 h; and as time passed, the granular green fluorescence gradually decreased.

Figure 7b,c show the growth curve of HepG2 cells in continuous culture for 72 h after different concentrations of sEVs were added. The relative confluence degree of 20 μg/ml of iPSCs‐EC1‐sEVs was significantly higher than the 10 μg/ml, 6 μg/ml and control group at 48 h (*P* < 0.05). However, sEVs concentrations of 10 μg/ml and 6 μg/ml did not result in any statistically significant differences compared with the control group.

Compared with blank and control group, the 20 ng/ml of sEVs extracted on day 1, day 3, and day 5 led to obvious proliferation of HepG2 cells at 12 h, 24 h, and 48 h (Figure 7d,e). At 24 h, and 48 h, the relative confluence degree was not significant different among the groups of sEVs extracted on day 1, day 3, and day 5, and it also was not significant different among the group of blank‐0.5% ED‐KSR‐20 ng/ml, the group of blank‐20% ED‐KSR‐100 ng/ml and Control (Figure 7f,g).

The gene expression levels of pluripotency markers in each group of HepG2 cells and sEVs continuously collected for 5 days were measured by qRT‐PCR. The results showed that sEVs contained a small amount of Nanog, Oct4 and SOX2 mRNA. Relative to iPSCs‐EC1 cells that were cultured in complete medium, the iPSCs‐EC1‐sEVs expression levels of Nanog, Oct4 and SOX2 were 0.024 ± 0.008, 0.015 ± 0.009, and 0.024 ± 0.009, respectively (Figure 7h; Figure S4). Relative to the iPSCs‐HF1 cells cultured in complete medium, the Nanog, Oct4 and SOX2 expression levels in the iPSCs‐HF1‐sEVs were 0.404 ± 0.047, 0.108 ± 0.023, and 0.076 ± 0.010, respectively (Figure 7i; Figure S4). The gene expression levels in each of the four groups of HepG2 cells were extremely low and did not exceed five‐thousandths of that of their respective control (Figure 7h,i; Figure S4).

These results indicate that HepG2 cells can endocytose sEVs into cells and localize to the cytoplasmic of the cell; when the sEVs reached a certain concentration, the promotion of HepG2 cell proliferation was statistically significant.

## 4 DISCUSSION

EVs play a role in transferring information during cell communication (Simons & Raposo, 2009). Studies have reported that stem cell‐derived EVs play an important role in human disease, and can also be used to solve problems that are associated with the use of stem cells in clinical application (Zhao et al., 2015). However, previous studies have not defined the most suitable collection conditions for hiPSCs culture supernatants. In this study, we aimed to establish suitable culture conditions to enable the collection of hiPSCs culture supernatant; we then used M‐UC and EQ to determine whether sEVs with biological activity could be extracted and thus provide the basis for the study of sEVs function.

To better study the biological functions of sEVs, we had to ensure that the sEVs donor cells grew optimally. Under normal culture conditions, hiPSCs‐EC1 are cultured in E8 complete medium, and hiPSCs‐HF1 cells are cultured in mTeSR1 complete medium. However, these two complete media are rich in proteins and cytokines (Chen et al., 2011), and ultracentrifugation may cause an excess of protein components to adhere to the sEVs during centrifugation (Tauro et al., 2012). Therefore, excessive protein content in the medium could affect the purity of the sEVs and affect subsequent experiments. Moreover, for economic reasons, neither mTeSR1 nor E8 is suitable for long‐term collection of cell culture supernatants. Gibco™ KnockOut™ Serum Replacement is a more defined, FBS‐free medium supplement that supports the growth of pluripotent stem cells cultured for 20 years, and it is the gold standard for feeder‐dependent iPSC culture, However, KSR cannot maintain the stemness of hiPSCs in the long‐term culture without MEF. In order to maintain the stemness of hiPSCs, we speculate that Matrigel Matrix contains extracellular matrix proteins, including Laminin, Collagen IV, heparin sulfate proteoglycans, entactin/nidogen, and a number of growth factors (TGF‐beta, epidermal growth factor, insulin‐like growth factor, fibroblast growth factor, tissue plasminogen activator, etc.), which acts on a part role of feeder cells. Furthermore, in our study hiPSCs do not need maintain the pluripotent and stemness of the cells for longer periods, while it could maintain the typical characteristics of stem cell for collection time (5 days) of sEVs. Therefore, KSR and Matrigel Matrix was chosen to prepare the extraction medium to minimize any impact on the sEVs collected. In addition, the ALP, immunofluorescence, flow cytometry and qRT‐PCR results also showed that both types of iPSCs could maintain their undifferentiated state with 5 days of collection in the production medium containing ED‐KSR; so, a well‐defined and economical KSR can be used as an additive to prepare extraction medium for 5 days of iPSCs culture supernatants. The results showed that 0.5% ED‐KSR had minimal interference with the extraction of sEVs and with subsequent experiments. In addition, we could not get some information about preparation process and components of KSR from the instructions or papers. And it is also not clear whether EVs in KSR. In order to avoid EVs or other EVs‐like particles contained in KSR that may affect the downstream experiment, it was ultracentrifugated at 120,000 × *g* for 18 h. Compared with the particle number of 20% KSR in D/F12 medium, after UC treatment, the 20% ED‐KSR was reduced to 10% of amount before treatment, while the 0.5% ED‐KSR could not even detect particles. Therefore, we speculated that EVs or other EVs‐like particles were removed after ultracentrifugation and optimal KSR concentration.

Next, we conducted a study of the days of collection of hiPSCs culture supernatants. It has previously been reported that when cells grow to an 80% density, complete medium should be replaced with extraction medium and culturing should continue for 48 h; then, the culture supernatant can be collected for the extraction of EVs (Hu et al., 2015). This approach can extract high‐quality EVs but may also waste donor cells. Therefore, in our study, to test whether the hiPSCs were undifferentiated and in a normal growth state, we continuously collected culture supernatants for 5 days and replaced the fresh EVs‐PM daily. On the one hand, although the viability and some stemness characteristics of hiPSCs can be remained after 5 days of continuous collection of the culture supernatants, the morphology and the SSEA4 and TRA‐1‐60 expression of hiPSCs changed gradually during 5 days, especially for hiPSCs‐HF1 on day 5. That means the status of cells were not completely consistent for 5 days under the culture condition. On the other hand, the results also show that the basic characteristics of sEVs are certain consistency despite some change in the status of cells, which suggest that the active, pure, sufficient sEVs can be extracted by this method for 5 days .Moreover, these sEVs further explore their biological function and survey their contents by multi‐omics study, which will be potential for clinical application. Therefore, it could provide a method to obtain a large number of sEVs for researchers. At the same time, using this method should be considered the status of cells during the culture period.

The literature shows that the gold standard method for EV isolation is ultracentrifugation (Li et al., 2017); however, other methods such as differential centrifugation (Wang et al., 2015) and PEG precipitation (Théry et al., 2006) have also been used. In these papers, the authors evaluated the ultracentrifugation method and the ExoQuick‐TC™ kit method, which were able to extract cell‐derived sEVs, but the authors also found an increased expression of the membrane protein Flotillin‐1, which may have occurred because the single ultracentrifugation method requires continuous centrifugation at different speeds, resulting in the destruction of the exosome membrane. In addition, sEVs extraction by the ExoQuick‐TC™ kit method also showed the presence of 200 nm particles and significant protein contamination (Lobb et al., 2015). Therefore, in our experiments, we improved the extraction medium for sEVs collection, and we also added a process of filtration and ultrafiltration for extraction instead of continuous centrifugation. Furthermore, our results show that this process can not only improve the damage of sEVs during the tedious centrifugation step, but it can also filter out other contaminant proteins and particles. Additional HepG2 proliferation experiments also showed that the optimized extraction method can result in biologically active sEVs, which lays a foundation for normal cell proliferation and the treatment of diseases such as liver failure.

WB also showed that the expression of CD63 was significantly higher in sEVs than in cells and that under the same loading conditions, CD63 was essentially undetectable in cells but was highly expressed in sEVs; these differences are likely because CD63, a member of the tetraspanin family (Hurwitz et al., 2018, Van Niel et al., 2011), plays an important role in the assembly of sEVs and is thus highly enriched in sEVs (Logozzi et al., 2009). Interestingly, the HSP70 protein was expressed in sEVs extracted via M‐UC but was not expressed in sEVs extracted via EQ. According to the literature, we hypothesized that the different extraction methods affected the enrichment of proteins in the sEVs (Shurtleff et al., 2016, Sinha et al., 2018). As previously reported in the literature, the sEVs extraction method will have an impact on sEVs marker proteins, as shown by the difference in the expression of TSG101 in sEVs that were extracted by different methods (Van Deun et al., 2014). Thus, we speculate that there is a similar explanation for the difference in Hsp70 expression in this experiment, but the specific reason remains to be verified. There may be some shortcomings of the EQ method such as protein damage, which will need to be experimentally verified.

## 5 CONCLUSION

The results show that 0.5% ED‐KSR is the optimal concentration for collecting the hiPSCs culture supernatant, and that the culture supernatant can be continuously collected for 5 days while maintaining high cell viability and some pluripotent characteristics. In the future study, we will further improve and optimize sEVs extraction method to ensure the characteristics of hiPSCs cells for certain periods. Both the M‐UC and EQ methods can extract biologically active sEVs from the culture supernatant under these conditions. Furthermore, the function of specific sEVs on recipient cells will be confirmed in future studies.

## CONFLICTS OF INTEREST

The authors report no conflicts of interest.

## AUTHOR CONTRIBUTIONS

Yingtang Gao, Tong Li, and Ying Luo conceived and planned the experiments; Ying Luo, Dunqin Gao, Cheng Lou, and Wenhui Niu performed the iPSCs characterization studies and exosome isolation and characterization; Peng Wang performed flow cytometry; and Dunqin Gao, Ying Luo, and Yingtang Gao analysed the data and wrote the manuscript. All authors read and approved the final manuscript.

## Supporting information

SUPPORTING INFORMATION

SUPPORTING INFORMATION

SUPPORTING INFORMATION

SUPPORTING INFORMATION

SUPPORTING INFORMATION

SUPPORTING INFORMATION

SUPPORTING INFORMATION

SUPPORTING INFORMATION

SUPPORTING INFORMATION

SUPPORTING INFORMATION

## References

[jev212065-bib-0009] Alderton, G. K. Metastasis. Exosomes drive premetastatic niche formation. Nat Rev Cancer. 2012;12:447. 22722393 10.1038/nrc3304

[jev212065-bib-0004] Amabile, G. , Meissner, A. Induced pluripotent stem cells: current progress and potential for regenerative medicine. Trends Mol Med. 2009;15:59‐68. 19162546 10.1016/j.molmed.2008.12.003

[jev212065-bib-0017] Baietti, M. F. , Zhang, Z. , Mortier, E. , Melchior, A. , Degeest, G. , Geeraerts, A. , Ivarsson, Y. , Depoortere, F. , Coomans, C. , Vermeiren, E. , Zimmermann, P. , David, G. Syndecan‐syntenin‐ALIX regulates the biogenesis of exosomes. Nat Cell Biol. 2012;14:677‐85. 22660413 10.1038/ncb2502

[jev212065-bib-0022] Chen, G. , Gulbranson, D. R. , Hou, Z. , Bolin, J. M. , Ruotti, V. , Probasco, M. D. , Smuga‐Otto, K. , Howden, S. E. , Diol, N. R. , Propson, N. E. , Wagner, R. , Lee, G. O. , Antosiewicz‐Bourget, J. , Teng, J. M. C. , Thomson, J. A. Chemically defined conditions for human iPSC derivation and culture. Nat Methods. 2011;8:424‐9. 21478862 10.1038/nmeth.1593 PMC3084903

[jev212065-bib-0003] Chin, M. H. , Mason, M. J. , Xie, W. , Volinia, S. , Singer, M. , Peterson, C. , Ambartsumyan, G. , Aimiuwu, O, Richter, L. , Zhang, J. , Khvorostov, I. , Ott, V. , Grunstein, M. , Lavon, N. , Benvenisty, N. , Croce, CM, Clark, A. T. , Baxter, T. , Pyle, A. D. , Teitell, M. A. Induced pluripotent stem cells and embryonic stem cells are distinguished by gene expression signatures. Cell stem cell. 2009;5:111‐23. 19570518 10.1016/j.stem.2009.06.008 PMC3448781

[jev212065-bib-0008] Colombo, M. , Raposo, G. , Théry, C. Biogenesis, secretion, and intercellular interactions of exosomes and other extracellular vesicles. Annu Rev Cell Dev Biol. 2014;30:255‐89. 25288114 10.1146/annurev-cellbio-101512-122326

[jev212065-bib-0014] Gao, W. , Liu, H. , Yuan, J. , Wu, C. , Huang, D. , Ma, Y. , Zhu, J. , Ma, L. , Guo, J. , Shi, H. , Zou, Y. , Ge, J. Exosomes derived from mature dendritic cells increase endothelial inflammation and atherosclerosisviamembrane TNF‐α mediated NF‐κB pathway. J Cell Mol Med. 2016;20:2318‐27. 27515767 10.1111/jcmm.12923 PMC5134386

[jev212065-bib-0010] Hendrix, A. , Hume, A. N. Exosome signaling in mammary gland development and cancer. Int J Dev Biol. 2011;55:879–87. 22161843 10.1387/ijdb.113391ah

[jev212065-bib-0024] Hu, G. W. , Li, Q. , Niu, X. , Hu, B. , Liu, J. , Zhou, S. M. , Guo, S. C. , Lang, H. L. , Zhang, C. Q. , Wang, Y. , Deng, Z. F. Exosomes secreted by human‐induced pluripotent stem cell‐derived mesenchymal stem cells attenuate limb ischemia by promoting angiogenesis in mice. Stem Cell Res Ther. 2015;6:10. 26268554 10.1186/scrt546 PMC4533800

[jev212065-bib-0029] Hurwitz, S. N. , Cheerathodi, M. R. , Nkosi, D. , York, S. B. , Meckes, D. G. Jr. Tetraspanin CD63 bridges autophagic and endosomal processes to regulate exosomal secretion and intracellular signaling of epstein‐barr virus LMP1. J Virol. 2018;92, e01969‐17. 29212935 10.1128/JVI.01969-17 PMC5809724

[jev212065-bib-0025] Li, P. , Kaslan, M. , Lee, S. H. , Yao, J. , Gao, Z. Progress in Exosome Isolation Techniques. Theranostics. 2017;7:789‐804. 28255367 10.7150/thno.18133 PMC5327650

[jev212065-bib-0019] Livak, K. J. , Schmittgen, T. D. Analysis of relative gene expression data using real‐time quantitative PCR and the 2(‐Delta DeltaC(T)) Method. Methods. 2001;25:402‐8. 11846609 10.1006/meth.2001.1262

[jev212065-bib-0028] Lobb, R. J. , Becker, M. , Wen, S. W. , Wong, C. S. , Wiegmans, A. P. , Leimgruber, A. , Möller, A. Optimized exosome isolation protocol for cell culture supernatant and human plasma. J Extracell Vesicles. 2015;4:27031. 26194179 10.3402/jev.v4.27031 PMC4507751

[jev212065-bib-0031] Logozzi, M. , De Milito, A. , Lugini, L. , Borghi, M. , Calabro, L. , Spada, M. , Perdicchio, M. , Marino, M. L. , Federici, C. , Iessi, E. , Brambilla, D. , Venturi, G. , Lozupone, F. , Santinami, M. , Huber, V. , Maio, M. , Rivoltini, L. , Fais, S. High levels of exosomes expressing CD63 and caveolin‐1 in plasma of melanoma patients. PloS one. 2009;4:e5219. 19381331 10.1371/journal.pone.0005219 PMC2667632

[jev212065-bib-0018] Luo, Y. , Lou, C. , Zhang, S. , Zhu, Z. , Xing, Q. , Wang, P. , Liu, T. , Liu, H. , Li, C. , Shi, W. , Du, Z. , Gao, Y. Three‐dimensional hydrogel culture conditions promote the differentiation of human induced pluripotent stem cells into hepatocytes. Cytotherapy. 2018;20:95‐107. 28969895 10.1016/j.jcyt.2017.08.008

[jev212065-bib-0006] Ratajczak, M. Z. , Jadczyk, T. , Pędziwiatr, D. , Wojakowski, W. New advances in stem cell research: practical implications for regenerative medicine. Pol Arch Med Wewn. 2014;124:417‐26. 24956404 10.20452/pamw.2355

[jev212065-bib-0032] Shurtleff, M. J. , Temoche‐Diaz, M. M. , Karfilis, K. V. , Ri, S. , Schekman, R. Y‐box protein 1 is required to sort microRNAs into exosomes in cells and in a cell‐free reaction. Elife. 2016; 5: e19276. 27559612 10.7554/eLife.19276 PMC5047747

[jev212065-bib-0020] Simons, M. , Raposo, G. Exosomes–vesicular carriers for intercellular communication. Curr Opin Cell Biol. 2009;21:575‐81. 19442504 10.1016/j.ceb.2009.03.007

[jev212065-bib-0033] Sinha, A. , Principe, S. , Alfaro, J. , Ignatchenko, A. , Ignatchenko, V. , Kislinger, T. Proteomic profiling of secreted proteins, exosomes, and microvesicles in cell culture conditioned media. Methods Mol Biol. 2018;1722:91‐102. 29264800 10.1007/978-1-4939-7553-2_6

[jev212065-bib-0011] Taheri, B. , Soleimani, M. , Fekri Aval, S. , Esmaeili, E. , Bazi, Z. , Zarghami, N. Induced pluripotent stem cell‐derived extracellular vesicles: a novel approach for cell‐free regenerative medicine. J Cell Physiol. 2019;234(6):8455–64. 30478831 10.1002/jcp.27775

[jev212065-bib-0001] Takahashi, K. , Tanabe, K. , Ohnuki, M. , Narita, M. , Ichisaka, T. , Tomoda, K. , Yamanaka, S. Induction of pluripotent stem cells from adult human fibroblasts by defined factors. Cell. 2007;131(5):861‐72. 18035408 10.1016/j.cell.2007.11.019

[jev212065-bib-0023] Tauro, B. J. , Greening, D. W. , Mathias, R. A. , Ji, H. , Mathivanan, S. , Scott, A. M. , Simpson, R. J. Comparison of ultracentrifugation, density gradient separation, and immunoaffinity capture methods for isolating human colon cancer cell line LIM1863‐derived exosomes. Methods. 2012;56:293‐304. 22285593 10.1016/j.ymeth.2012.01.002

[jev212065-bib-0012] Théry, C. , Witwer, K. W. , Aikawa, E. , Alcaraz, M. J. , Anderson, J. D. , Andriantsitohaina, R. , Antoniou, A. , Arab, T. , Archer, F. , Atkin‐Smith, G. K. , Ayre, D. C. , Bach, J. M. , Bachurski, D. , Baharvand, H. , Balaj, L. , Baldacchino, S. , Bauer, N. N. , Baxter, A. A. , Bebawy, M. , Beckham, C. Minimal information for studies of extracellular vesicles 2018 (MISEV2018): a position statement of the International Society for Extracellular Vesicles and update of the MISEV2014 guidelines. J Extracell Vesicles. 2018;7(1):1535750. 30637094 10.1080/20013078.2018.1535750 PMC6322352

[jev212065-bib-0027] Théry, C. , Amigorena, S. , Raposo, G. , Clayton, A. Isolation and characterization of exosomes from cell culture supernatants and biological fluids. Curr Protoc Cell Biol. 2006;Chapter 3:Unit 3.22. 10.1002/0471143030.cb0322s30 18228490

[jev212065-bib-0034] Van Deun, J. , Mestdagh, P. , Sormunen, R. , Cocquyt, V. , Vermaelen, K. , Vandesompele, J. , Bracke, M. , De Wever, O. , Hendrix, A. The impact of disparate isolation methods for extracellular vesicles on downstream RNA profiling. J Extracell Vesicles. 2014;3. 10.3402/jev.v3.24858 PMC4169610 25317274

[jev212065-bib-0030] Van Niel, G. , Charrin, S. , Simoes, S. , Romao, M. , Rochin, L. , Saftig, P. , Marks, M. S. , Rubinstein, E. , Raposo, G. The tetraspanin CD63 regulates ESCRT‐independent and ‐dependent endosomal sorting during melanogenesis. Dev Cell. 2011;21:708‐21. 21962903 10.1016/j.devcel.2011.08.019 PMC3199340

[jev212065-bib-0005] Volarevic, V. , Markovic, B. S. , Gazdic, M. , Volarevic, A. , Jovicic, N. , Arsenijevic, N. , Armstrong, L. , Djonov, V. , Lako, M. , Stojkovic, M. Ethical and safety issues of stem cell‐based therapy. Int J Med Sci. 2018;15:36‐45. 29333086 10.7150/ijms.21666 PMC5765738

[jev212065-bib-0016] Wagner, K. , Welch, D. Feeder‐free adaptation, culture and passaging of human IPS cells using complete Knockout Serum Replacement feeder‐free medium. J Vis Exp. 2010;(41):2236. 20644503 10.3791/2236 PMC3157883

[jev212065-bib-0026] Wang, Y. , Zhang, L. , Li, Y. , Chen, L. , Wang, X. , Guo, W. , Zhang, X. , Qin, G. , He, S. H. , Zimmerman, A. , Liu, Y. , Kim, I. M. , Weintraub, N. L. , Tang, Y. Exosomes/microvesicles from induced pluripotent stem cells deliver cardioprotective miRNAs and prevent cardiomyocyte apoptosis in the ischemic myocardium. Int J Cardiol. 2015;192:61‐9. 26000464 10.1016/j.ijcard.2015.05.020 PMC4469495

[jev212065-bib-0015] Ye, S. B. , Li, Z. L. , Luo, D. H. , Huang, B. J. , Chen, Y. S. , Zhang, X. S. , Cui, J. , Zeng, Y. X. , Li, J. Tumor‐derived exosomes promote tumor progression and T‐cell dysfunction through the regulation of enriched exosomal microRNAs in human nasopharyngeal carcinoma. Oncotarget. 2014;5:5439‐52. 24978137 10.18632/oncotarget.2118 PMC4170615

[jev212065-bib-0007] Yeung, V. , Webber, J. P. , Dunlop, E. A. , Morgan, H. , Hutton, J. , Gurney, M. , Jones, E. , Falcon‐Perez, J. , Tabi, Z. , Errington, R. , Clayton, A. Rab35‐dependent extracellular nanovesicles are required for induction of tumour supporting stroma. Nanoscale. 2018;10(18):8547‐8559. 29693684 10.1039/c8nr02417k

[jev212065-bib-0002] Yu, J. , Vodyanik, M. A. , Smuga‐Otto, K. , Antosiewicz‐Bourget, J. , Frane, J. L. , Tian, S. , Nie, J. , Jonsdottir, G. A. , Ruotti, V. , Stewart, R. , Slukvin, I. I. , Thomson, J. A. Induced pluripotent stem cell lines derived from human somatic cells. Science. 2007;318:1917‐20. 18029452 10.1126/science.1151526

[jev212065-bib-0021] Zhao, Y. , Sun, X. , Cao, W. , Ma, J. , Sun, L. , Qian, H. , Zhu, W. , Xu, W. Exosomes derived from human umbilical cord mesenchymal stem cells relieve acute myocardial ischemic injury. Stem Cells Int. 2015;2015:761643. 26106430 10.1155/2015/761643 PMC4461782

[jev212065-bib-0013] Zhu, Y. , Wang, Y. , Zhao, B. , Niu, X. , Hu, B. , Li, Q. , Zhang, J. , Ding, J. , Chen, Y. , Wang, Y. Comparison of exosomes secreted by induced pluripotent stem cell‐derived mesenchymal stem cells and synovial membrane‐derived mesenchymal stem cells for the treatment of osteoarthritis. Stem Cell Res Ther. 2017;8:64. 28279188 10.1186/s13287-017-0510-9 PMC5345222

