# Supplementary figures and images for: Optimized culture methods for isolating small extracellular vesicles derived from human induced pluripotent stem cells
Source: J Extracell Vesicles. 2021 Apr 10;10(6):e12065. doi: 10.1002/jev2.12065 (PMC8035677; doi:10.1002/jev2.12065)

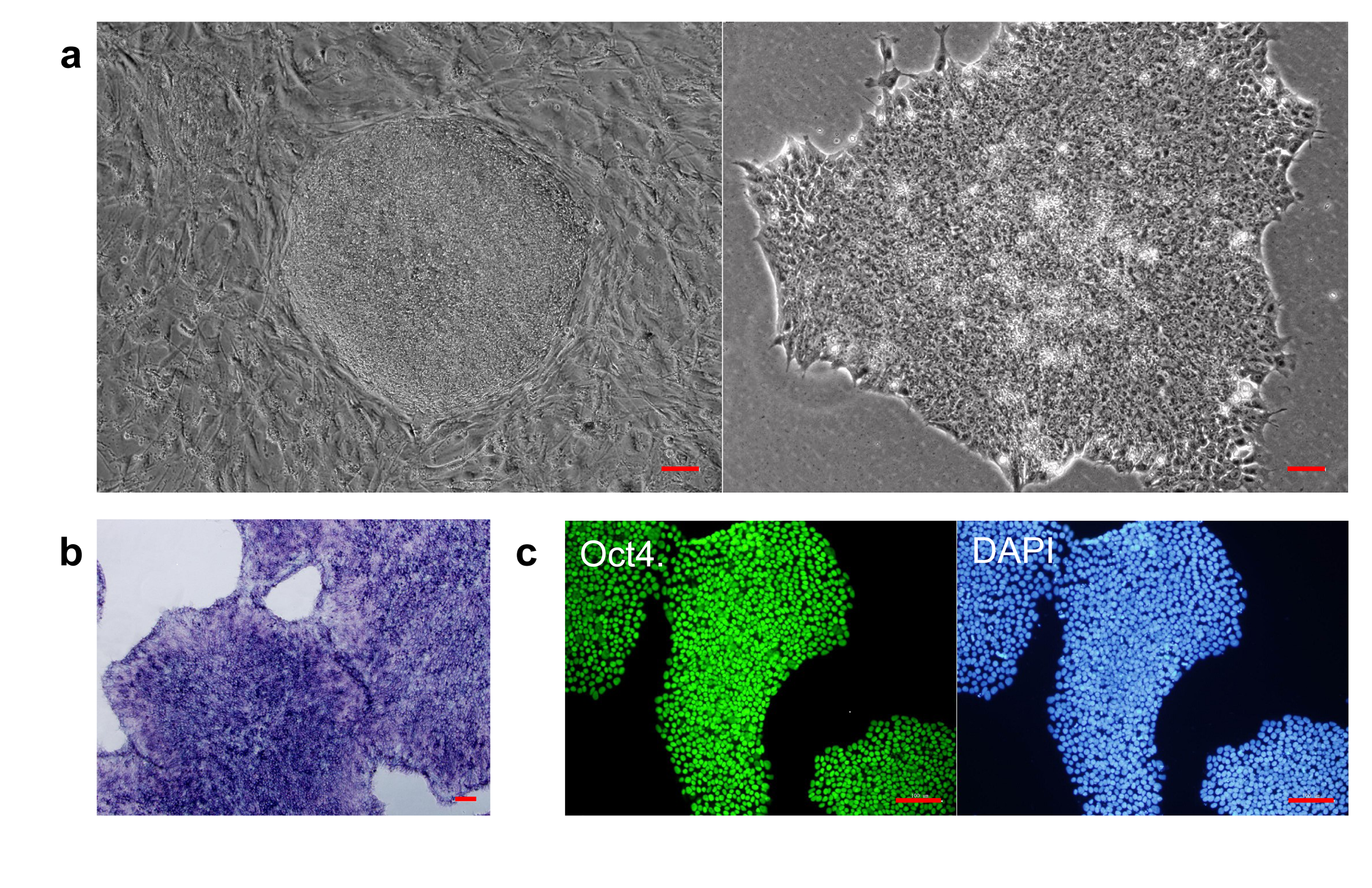

Supplement: Supplementary file 1 — SUPPORTING INFORMATION [file JEV2-10-e12065-s008.tif]

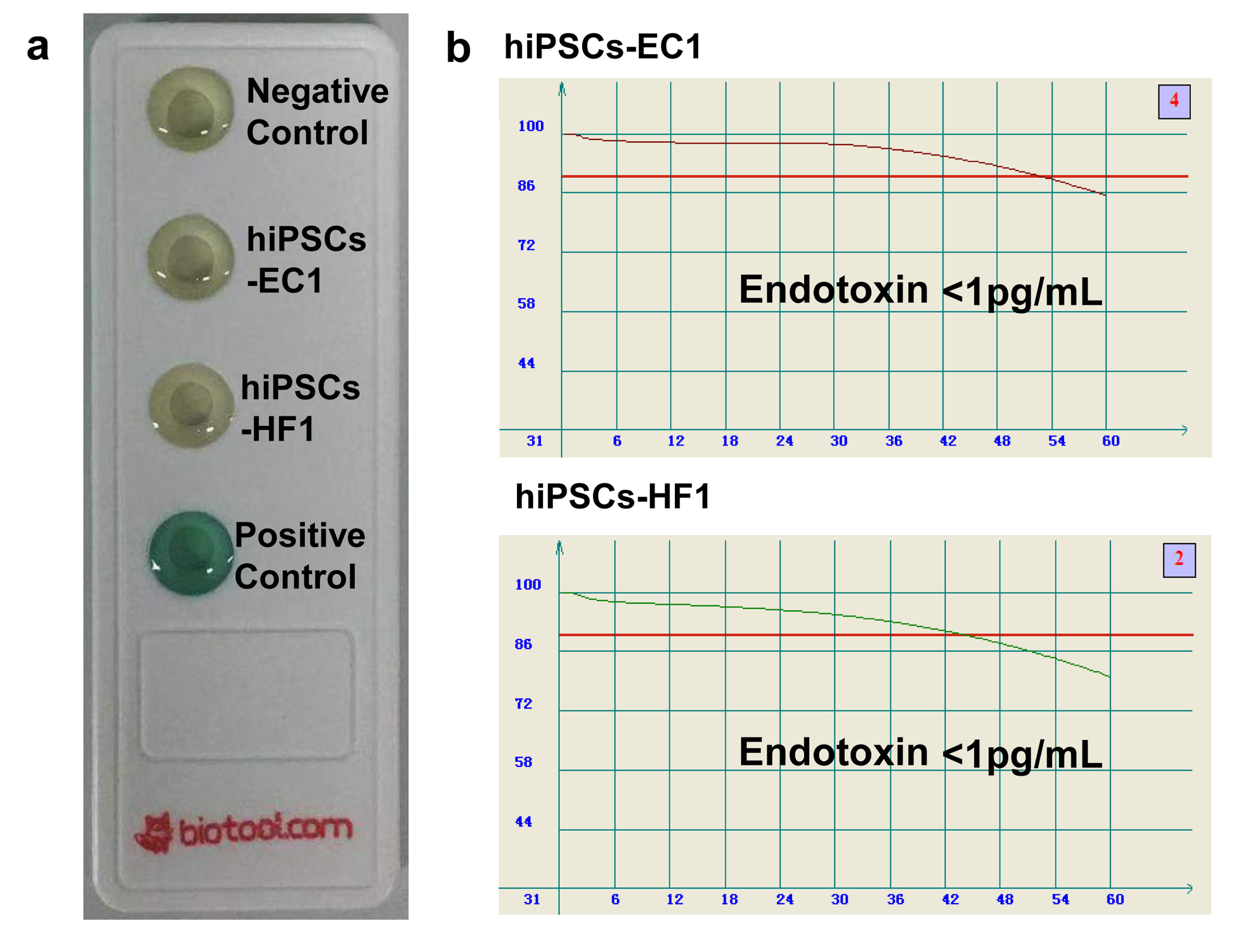

Supplement: Supplementary file 2 — SUPPORTING INFORMATION [file JEV2-10-e12065-s009.tif]

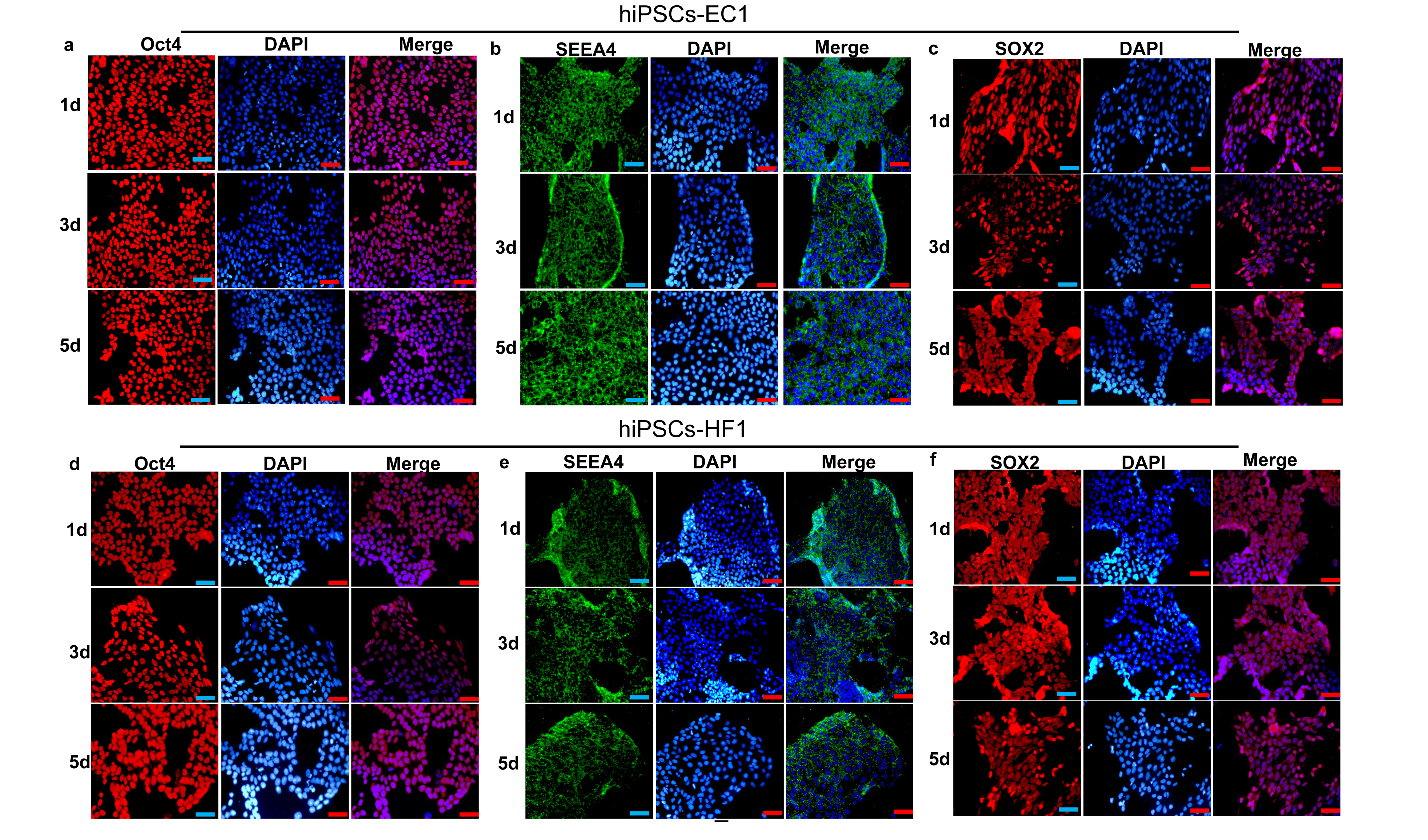

Supplement: Supplementary file 3 — SUPPORTING INFORMATION [file JEV2-10-e12065-s004.tif]

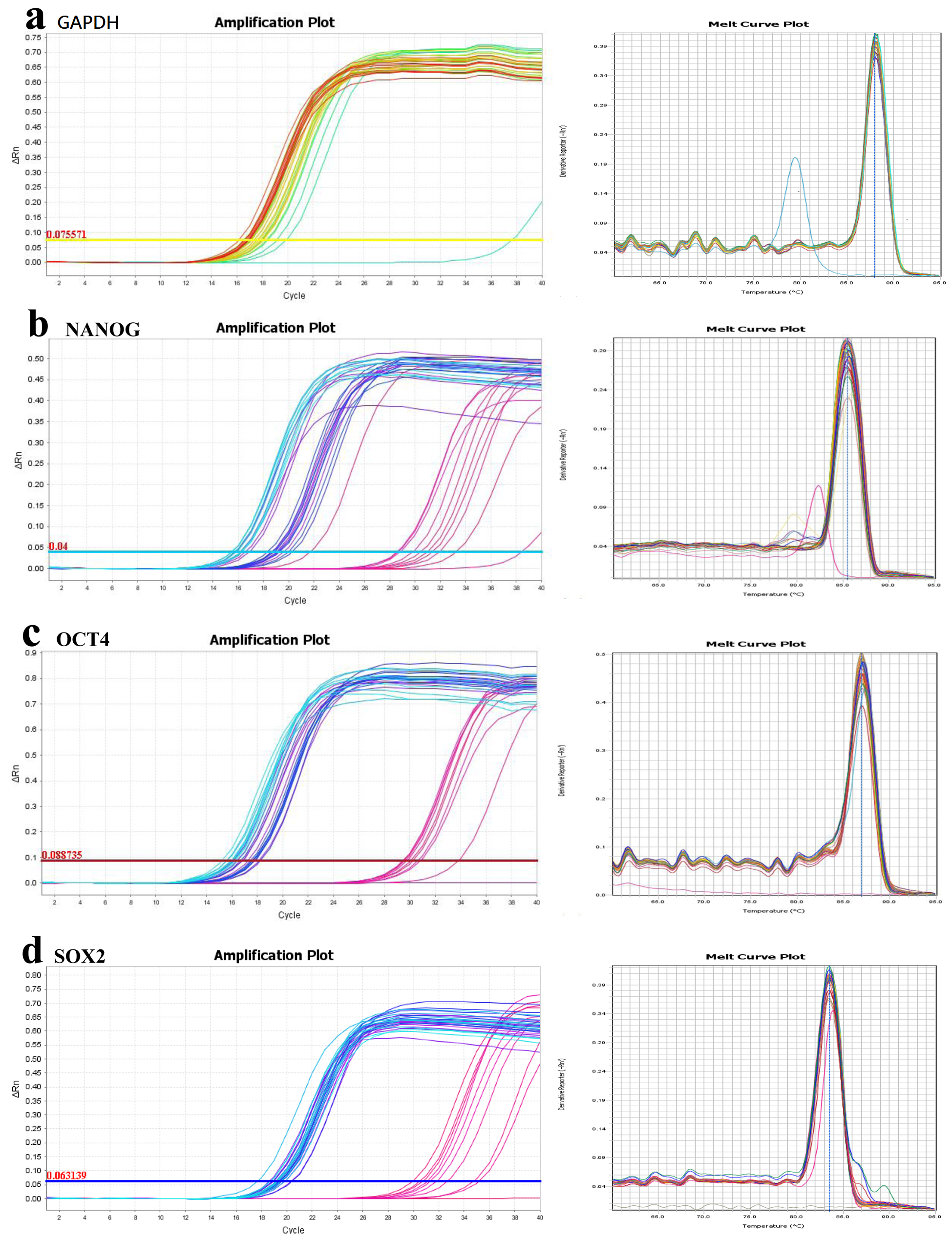

Supplement: Supplementary file 4 — SUPPORTING INFORMATION [file JEV2-10-e12065-s003.tif]

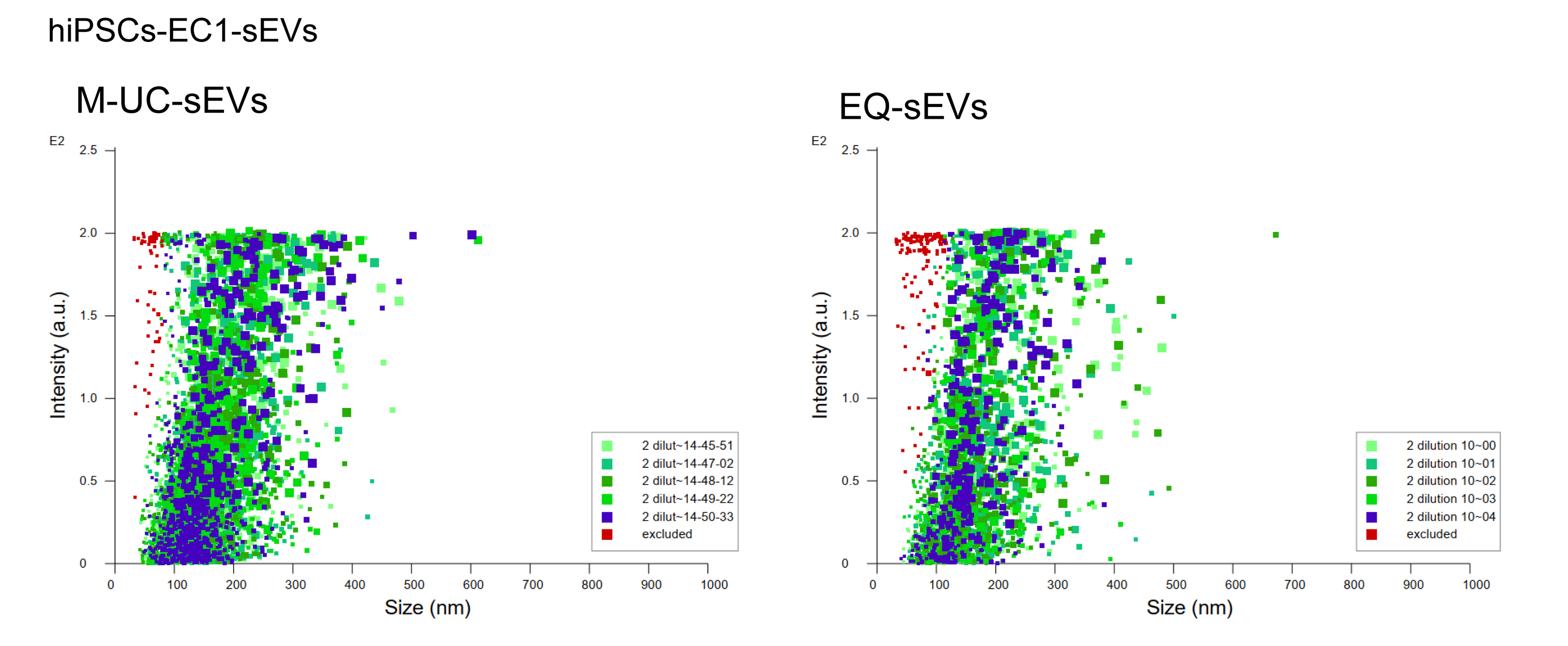

Supplement: Supplementary file 5 — SUPPORTING INFORMATION [file JEV2-10-e12065-s005.tif]

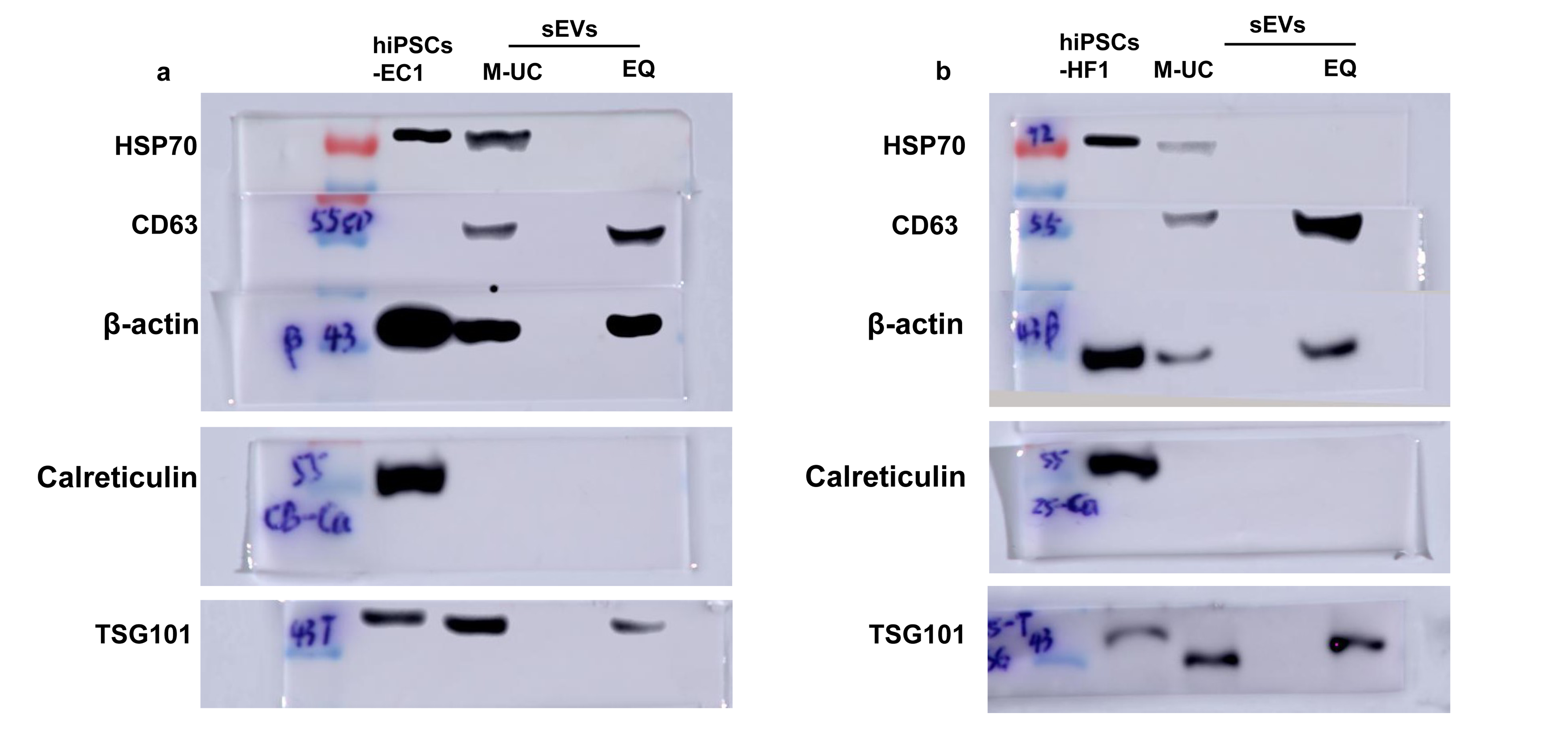

Supplement: Supplementary file 6 — SUPPORTING INFORMATION [file JEV2-10-e12065-s007.tif]

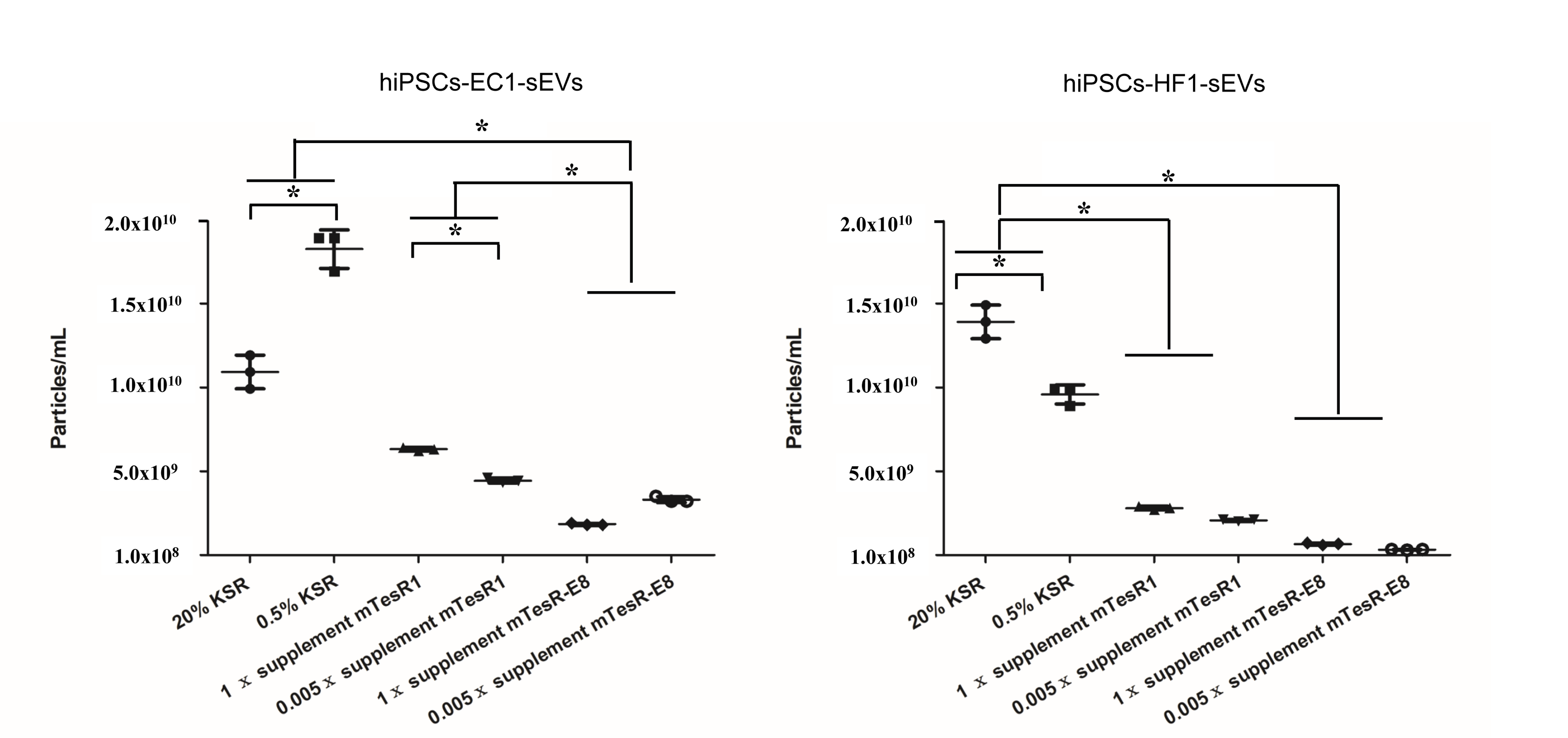

Supplement: Supplementary file 7 — SUPPORTING INFORMATION [file JEV2-10-e12065-s002.tif]
