# Supplementary material for: Optimized culture methods for isolating small extracellular vesicles derived from human induced pluripotent stem cells
Source: J Extracell Vesicles. 2021 Apr 10;10(6):e12065. doi: 10.1002/jev2.12065 (PMC8035677; doi:10.1002/jev2.12065)
Supplement: Supplementary file 8 — SUPPORTING INFORMATION [file JEV2-10-e12065-s006.docx]

**Supplement Table 1. Antibody information**

| **Antibody** | **Clonality** | **Species** | **Cat. No** | **clone name** | **Tested Application** | **Dilution** | **Source** | **reducing/non-reducing conditions** |
| --- | --- | --- | --- | --- | --- | --- | --- | --- |
| Anti-Oct4 | Polyclonal | Rabbit | ab109884^#^ (ab19857) | – | IF | 1:200 | Abcam, Inc. | – |
| Anti-SOX2 | Polyclonal | Rabbit | ab109884  (ab97959) | – | IF | 1:200 | Abcam, Inc. | – |
| Anti-SSEA4 | Monoclonal | Mouse | ab109884  (ab16287) | MC813 | IF | 1:100 | Abcam, Inc. | – |
|  |  |  |  |  | Flow Cyt | 1:100 |  |  |
| Anti-Tra-1-60 | Monoclonal | Mouse | ab109884 (ab16288) | TRA-1-60 | Flow Cyt | 1:200 | Abcam, Inc. | – |
| Anti-Calreticulin | Monoclonal | Rabbit | ab92516 | EPR3924 | WB | 1:1000 | Abcam, Inc. | **reducing** |
| Anti-CD63 | Polyclonal | Rabbit | EXOAB-CD63A-1 | – | WB | 1:1000 | System Biosciences (SBI), Inc. | **reducing** |
| Anti-HSP70 | Polyclonal | Rabbit | EXOAB-Hsp70A-1 | – | WB | 1:1000 | SBI, Inc. | **reducing** |
| Anti-TSG101 | Monoclonal | Rabbit | ab125011 | EPR7130(B) | WB | 1:1000 | Abcam, Inc. | **reducing** |
| Anti-β-actin | Monoclonal | Mouse | ab8226 | mAbcam 8226 | WB | 1:1000 | Abcam, Inc | **reducing** |

^#^ Embryonic Stem Cell Marker Panel (ab109884) (Human: Oct4，Nanog，Tra-1-60，SOX2，SSEA4)
