# Supplementary material for: Optimized culture methods for isolating small extracellular vesicles derived from human induced pluripotent stem cells
Source: J Extracell Vesicles. 2021 Apr 10;10(6):e12065. doi: 10.1002/jev2.12065 (PMC8035677; doi:10.1002/jev2.12065)

**人诱导多能干细胞（iPSC）质检报告**

**Report on Quality Assay of Human Induced pluripotent Stem Cells (iPSC)**

赛贝生物（Cellapy®）提供的人诱导多能干细胞（hiPSC）是由人体细胞通过非整合的方法诱导获得。hiPSC在无饲养层、化学成分明确、并且无动物源成分的PSCeasy®人多潜能干细胞培养体系中培养，适合临床级的干细胞研究和应用。hiPSC在PSCeasy®人多潜能干细胞培养体系中可以长期稳定快速增殖，具有高度近似hESC的克隆形态和基因表达，长期维持正常核型，并在体内外具有三胚层分化潜能。

Human induced pluripotent stem cells (hiPSC) provided by Cellapy are derived from human cells by non-integrating method. HiPSC is cultured in PSCeasy® human pluripotent stem cell culture system without feeder cells, clear chemical composition, and without serum or ingredients of animal origin. It is suitable for clinical stem cell research and application. HiPSC can proliferate steadily and rapidly for a long time in the PSCeasy® culture system. It has a highly similar clonal morphology and gene expression of hESC, maintains normal karyotype for a long time, and has the potential of triembryonic differentiation in vivo and in vitro.

**细胞信息：Cell information: (Cat No.** **CA4024106)**

| **项目 Project** | **内容 Content** |
| --- | --- |
| 名称 Name | hiPSC-人诱导多能干细胞 Human induced pluripotent stem cells (p22) |
| 来源 Source | 37岁男性尿液肾上皮细胞 Urine renal epithelial cells in 37-year-old male |
| 诱导方式 Induction method | 仙台 Sendai virus |
| 规格 Specification | 1×10^6^ |

**生长曲线检测结果：**细胞增殖良好，倍增时间为24小时，为快速增长型细胞。

**The results of growth curve** showed that the cells proliferated well and the doubling time was 24 hours.


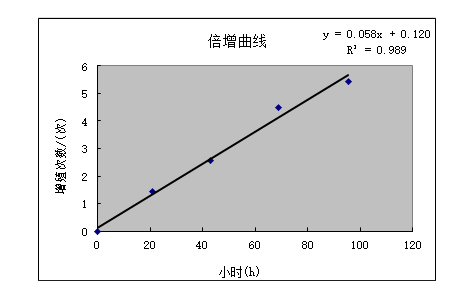

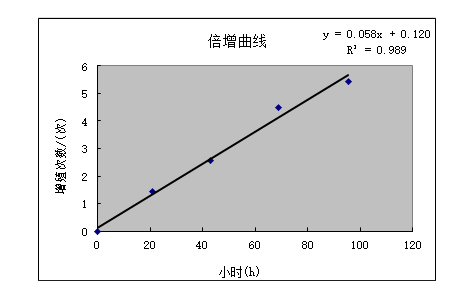


注：细胞在一次传代内的生长曲线与倍增曲线。A：细胞培养4天的生长曲线。纵轴：细胞数，横轴：培养时间。B：细胞培养4天的倍增曲线。纵轴：细胞倍增次数，横轴：培养天数。

Note: Cell growth curve and multiplication curve in one passage. A: Growth curve of cells cultured for 4 days. Y axis: number of cells, X axis: culture time. B: Multiplication curve of cell culture for 4 days. Y axis: number of cell doubling, X axis: days of culture.

**真菌和细菌污染检测：无真菌或细菌的污染 支原体污染检测：无支原体污染**

| **Detection of fungal and bacterial contamination: no fungal or bacterial contamination** | **Mycoplasma contamination detection:**  **no mycoplasma contamination** |
| --- | --- |


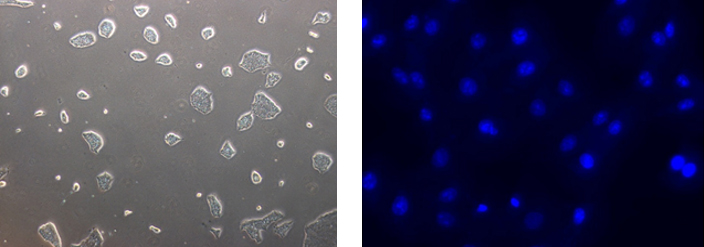


| 细胞复苏后培养24小时，光学显微镜下观察，无真菌或细菌的污染。  The cells were cultured 24 hours after resuscitation and observed under optical microscope. No fungi or bacterial contamination was found. | 吸取上清液，培养Vero细胞，通过DNA染色法检测，仅观察到细胞的DNA，并无支原体DNA的存在。  Vero cells were cultured with supernatant and detected by DNA staining. Only cell DNA was observed, and no Mycoplasma DNA was found. |
| --- | --- |

**免疫荧光染色检测： Immunofluorescence staining**

**
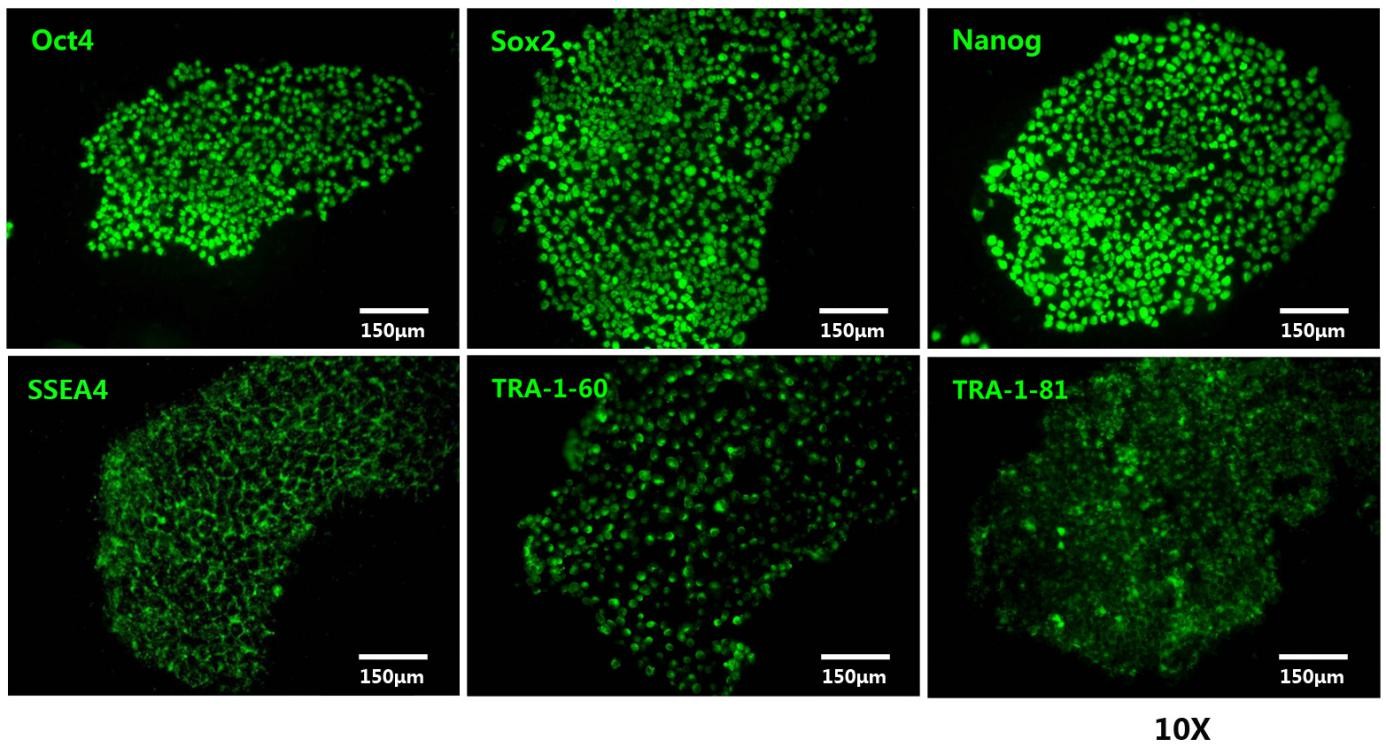
**

**细胞核型检测：Karyotype detection**


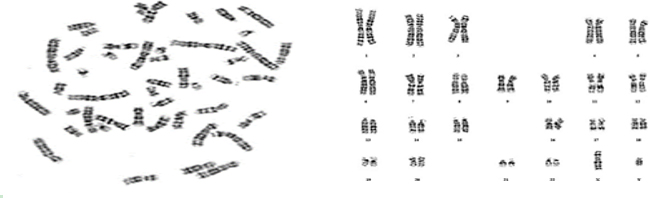


细胞核型检测--- 46，XY（G带），核型正常

Karyotype detection - - 46, XY (G band), normal karyotype.

**畸胎瘤检测：Detection of Teratoma**


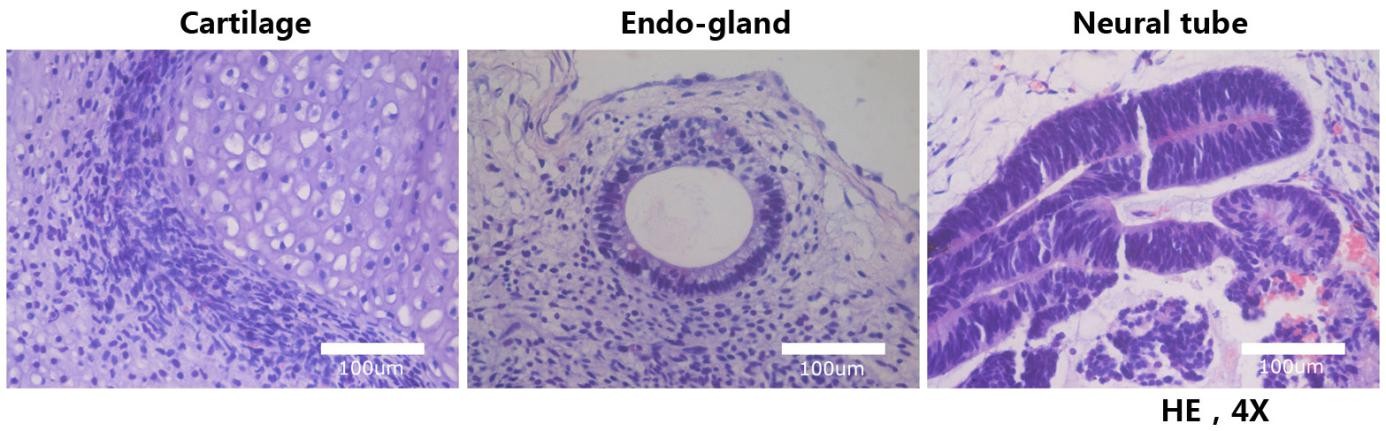

Supplement: Supplementary file 9 — SUPPORTING INFORMATION [file JEV2-10-e12065-s001.docx]
