# Supplementary material for: Optimized culture methods for isolating small extracellular vesicles derived from human induced pluripotent stem cells
Source: J Extracell Vesicles. 2021 Apr 10;10(6):e12065. doi: 10.1002/jev2.12065 (PMC8035677; doi:10.1002/jev2.12065)
Supplement: Supplementary file 10 — SUPPORTING INFORMATION [file JEV2-10-e12065-s010.docx]

**Change in Authorship Form**

Manuscript number: ZJEV-2019-0048R1

Manuscript title: Optimized culture methods for isolating small extracellular vesicles derived from

human induced pluripotent stem cells

☑ There is a change in the order of authorship.

|  | **Former Authorship** | **New Authorship** |
| --- | --- | --- |
| Name 1 | Dunqin Gao | Ying Luo |
| Name 2 | Ying Luo | Dunqin Gao |
| Name 3 | Peng Wang | Peng Wang |
| Name 4 | Cheng Lou | Cheng Lou |
| Name 5 | Tong Li | Tong Li |
| Name 6 | Wenhui Niu | Wenhui Niu |
| Name 7 | Yingtang Gao | Yingtang Gao |

**Authorizing Signatures**:

|  | **New Authorship** | **Signature** |
| --- | --- | --- |
| Name 1 | Ying Luo | 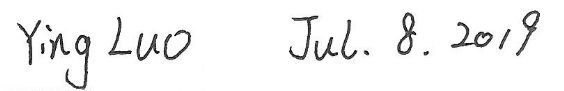 |
| Name 2 | Dunqin Gao | 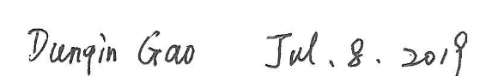 |
| Name 3 | Peng Wang | 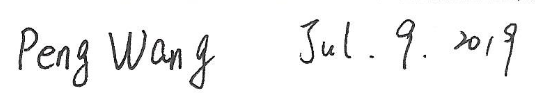 |
| Name 4 | Cheng Lou | 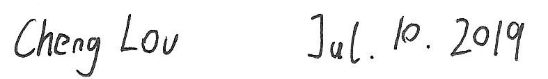 |
| Name 5 | Tong Li | 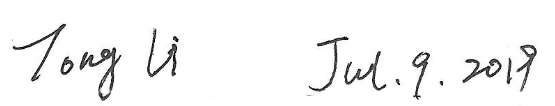 |
| Name 6 | Wenhui Niu | 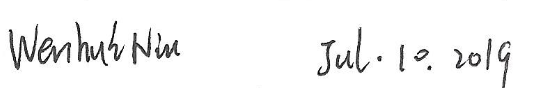 |
| Name 7 | Yingtang Gao | 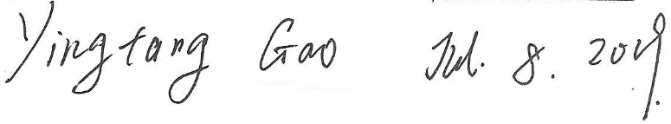 |
